# Aromatic Amines in Organic Synthesis. Part II. *p*-Aminocinnamaldehydes

**DOI:** 10.3390/molecules26144360

**Published:** 2021-07-19

**Authors:** Marek Pietrzak, Beata Jędrzejewska

**Affiliations:** Faculty of Chemical Technology and Engineering, UTP University of Sciences and Technology, Seminaryjna 3, 85-326 Bydgoszcz, Poland; beata@utp.edu.pl

**Keywords:** aromatic aldehydes, *p*-aminocinnamaldehyde, extended bond system

## Abstract

Ten derivatives of *p*-aminocinnamic aldehydes were prepared from the reaction of either aromatic amines with dimethylaminoacrolein or benzaldehydes with acetaldehyde. Their chemical structure and purity were verified by ^1^H NMR, ^13^C NMR and IR spectroscopic methods. We found that the synthesis applying dimethylaminoacrolein as the reagent gets better yields than the one based on the reaction with acetaldehyde. The yields of the cinnamic aldehydes varied according to the type of the amino group and the number and position of the substituents. The basic spectroscopic properties of the *p*-aminocinnamic aldehydes are also described since the compounds may be a precursor for the synthesis of dyes for diverse applications, e.g., in medicine and optoelectronics.

## 1. Introduction

Organic compounds containing different amino groups (or their derivatives) have attracted remarkable attention by virtue of their giant potential as dyes [1], solar cells [2,3], spectroscopic probes [4,5,6], one- [7,8] and multi-photon [9] polymerization initiators and drugs [10]. They have been studied for applications in various areas of photochemistry due to their structure versatility and facile modifications for electron-donating abilities [11,12,13]. In general, organic compounds bearing an alkylamino group may be synthesized through a related synthetic strategy using one of the following: Heck reaction [14], Knoevenagel condensation [13,15], Wittig reaction [16], Suzuki-Miyaura cross-coupling reaction [17] and several different reagents, such as aryl bromides with amino substituents [14], *p*-aminobenzaldehydes [13,15] and *p*-aminobenzoic acid derivatives [18,19].

Alternatively, organic compounds with an amino group, of particular interest for their nonlinear optical properties useful in modern technology, can be obtained using precursors with extended π-systems [20]. For the preparation of such compounds, standard *p*-aminobenzaldehydes should be replaced with *p*-aminocinnamaldehydes, their acids or esters [21,22].

There are different methods for the synthesis of cinnamaldehydes. One of them is based on the reaction between acetaldehyde with benzaldehydes having an electron-withdrawing substituent in the presence of KOH as a base [23]. The method is relatively simple and gives good yields. However, in the case of benzaldehydes with electron-donating substituents, in particular amino groups, this reaction has to be performed in concentrated sulfuric acid, and more drastic conditions are required. Moreover, products are isolated at a lower yield [23]. Therefore, alternative methods of synthesizing *p*-aminocinnamaldehydes with higher yields are sought. They can be obtained following the Heck reaction [24] or the reaction of aromatic amines with dimethylaminoacrolein [25].

**Scheme 1:**
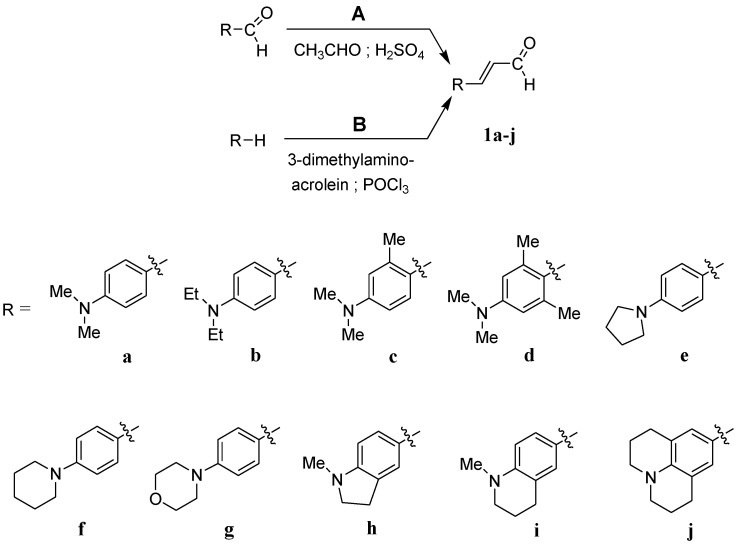
General routes for the preparation of derivatives of *p*-aminocinnamic aldehyde.

As part of our study on dyes with enlarged double bond systems that may be used as two-photon absorbers, [20] an optical amplifier, generator and modulator based on third order nonlinear optical phenomenon [26], photoinitiators of polymerization [8] or spectroscopic probes [6]. In the paper, we report synthesis, characterization and photophysical properties of the ten representatives of *p*-aminocinnamic aldehydes. They were obtained through two different procedures using corresponding benzaldehydes (Method A) or amines (Method B) as the starting materials (see Scheme 1). Method A is a single-step acid-catalyzed aldol condensation between acetaldehyde and appropriate benzaldehyde. This is a well-known method with moderate yields. Method B is analogous to the classical Vilsmeier–Haack formylation method; only 3-dimethylaminoacrolein is used instead of dimethylformamide. The mechanism of this reaction has been described by Ulrich and Brenmeier [25]. In this case, an electrophilic substitution takes place, and therefore, aromatic amines react readily.

## 2. Results

The synthetic routes for the preparation of ten *p*-aminocinnamic aldehydes are shown in Scheme 1. Six of these aldehydes (1c, 1d, 1e, 1f, 1h, 1i) have not been described in the literature yet. According to Method A, treatment of *p*-aminobenzaldehyde with acetaldehyde in concentrated sulfuric acid at a lower temperature (0 °C) followed by neutralization, afforded *p*-aminocinnamic aldehyde a good yield (32–54%). The yield of the synthesis was improved by ca. 6–16% for the reaction between aromatic amine and 3-dimethylaminoacrolein, in the presence of POCl_3_ conducted in chloroform at −20–0 °C (Method B). What is more, 4-(N,N-dimethylamino)-2,6-dimethylcinnamaldehyde (1d) can be obtained at a satisfactory yield only using Method B. This is probably due to the steric hindrance.

The structure and purity of all synthesized compounds were confirmed by spectroscopic methods, including NMR and IR spectra analysis (spectra are shown in the ESI file), as well as the elemental analysis. The NMR spectra confirmed the presence of signals from all protons and carbons in the synthesized molecules, as shown in the Experimental Section. For example, the proton signal of the aldehyde group is clearly visible at ca. 9.5 ppm, whereas the characteristic signals of the methine groups are given in the range from 7.8 to 6.2 ppm. The coupling constant for these protons (^3^*J*_H,H_ = 16 Hz) indicates that the compounds are in the *trans* configuration. The signal of carbon from the aldehyde group occurs at ca. 193–195 ppm and is shifted by about 0.7 ppm compared to the analogous benzaldehydes. The presence of the carbonyl group is also confirmed by the characteristic, strong signal in the IR spectrum in the frequency range from 1667 (1e) to 1649 cm^−1^ (1b).

**Figure 1:**
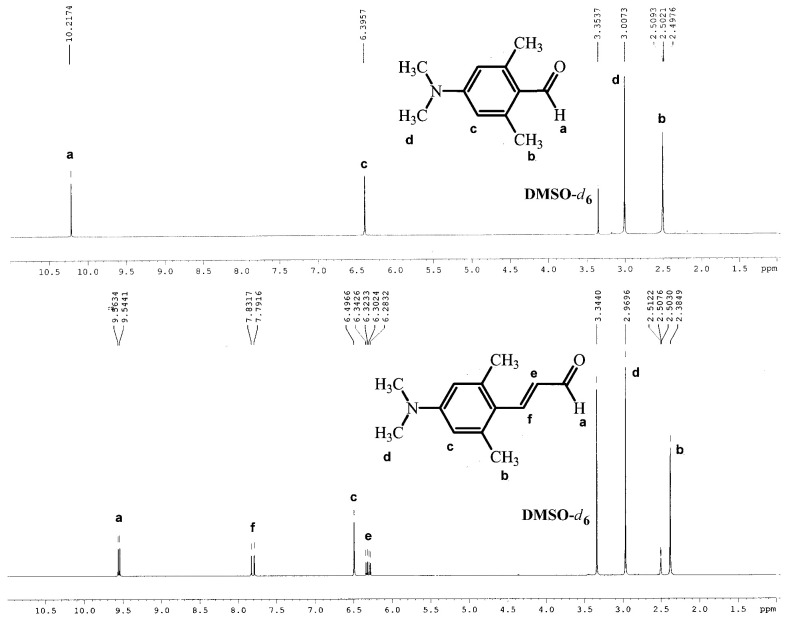
^1^H NMR spectra of 4-(dimethylamino)-2,6-dimethylbenzaldehyde and 4-(dimethylamino)-2,6-dimethylcinnamaldehyde (1d) in DMSO-*d*_6_.

Figure 1 illustrates the ^1^H NMR spectra of 4-(dimethylamino)-2,6-dimethylcinnamaldehyde (1d) in DMSO-*d*_6_ and its corresponding benzaldehyde to show differences in signal positions associated with elongation of the π-system.

The comparative analysis of the ^1^H NMR spectra of the 4-(dimethylamino)-2,6-dimethylbenzaldehyde and 4-(dimethylamino)-2,6-dimethylcinnamaldehyde (Figure 1) shows that the signal of the CHO group is shifted from 10.2 to 9.5 ppm and split into doublet with a coupling constant of 8.0 Hz. Additionally, in cinnamic aldehyde, there are two characteristic signals from methine hydrogen at ca. 7.8 and 6.3 ppm, with the *H*^e^ signal visible as a doublet of doublets with unequal coupling constants.

**Figure 2:**
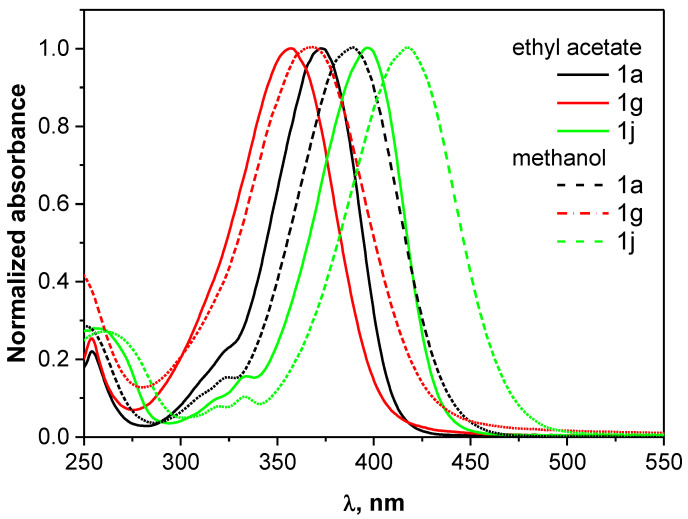
Normalized electronic absorption spectra of selected *p*-aminocinnamaldehydes in ethyl acetate and methanol.

Since the compounds can be used for the preparation of different groups of dyes, their basic spectroscopic data are compiled in Table 1. They are determined in two solvents of varying properties, i.e., methanol and ethyl acetate. The normalized electronic absorption spectra of selected *p*-aminocinnamic aldehydes (1a, 1g and 1j) in ethyl acetate (EtOAc) and methanol (MeOH) are shown in Figure 2, whereas the spectra for all synthesized compounds are presented in the ESI file.

The UV–Vis absorption spectra display one absorption peak at ca. 390–400 nm, attributed to the π→π* transition. Compound 1j exhibits the most redshifted absorption band with a maximum at 417.5 and 460 nm in methanol and ethyl acetate, respectively, compared to the parent 1a cinnamic aldehyde (maximum at 389 and 372.5 nm). The significant redshift is attributed to the presence of the stiffening alkylamino group, which enhanced the extent of electron delocalization over the whole molecule. On the other hand, the strongest hypsochromic effect is observed in cinnamaldehyde with morpholine residue. The presence of the oxygen atom in the six-membered piperidine ring probably causes the coplanar alignment of the molecule, which results in a higher energy barrier for the S_0_-S_1_ transition and shifting the absorption maximum to the blue region. The molar extinction coefficients of the *p*-aminocinnamic aldehydes are in the range from 28,900 to 45,700 M^−1^cm^−1^ and 31,400 to 44,500 M^−1^cm^−1^ in MeOH and EtOAc, respectively. These values are rather high, indicating that the compounds have a good light-harvesting ability.

The fluorescence spectra of the compounds 1a–j (exited at 370 and 390 nm in EtOAc and MeOH, respectively) showed a sharp band in the range from 440 to 480 nm (see Figures S3 and S4 in ESI), corresponding to blue light emission. Similar to the UV-Vis absorption, the fluorescence maximum of 1j is redshifted by 14 and 23 nm in MeOH and EtOAc, respectively, relative to the parent *p*-N,N-dimethylaminocinnamic aldehyde, which arises from the different donating ability of the amino group. The bathochromic shift of the absorption and fluorescence spectra also occurs with increasing solvent polarity. However, the change of ethyl acetate to methanol causes a small (approx. 15 nm) redshift of the absorption maximum. A more pronounced shift with increasing solvent polarity (ca. 25–40 nm) is observed for the fluorescence band, which indicates better stabilization of the excited state of the compounds in a more polar environment and suggests an increase of the dipole moment upon excitation. The fluorescence quantum yield of the compounds is small and does not exceed 2% in ethyl acetate and 0.5% in methanol, which suggests that the deactivation of the S_1_ excited-state occurs mostly by non-radiative processes.

**Figure 3:**
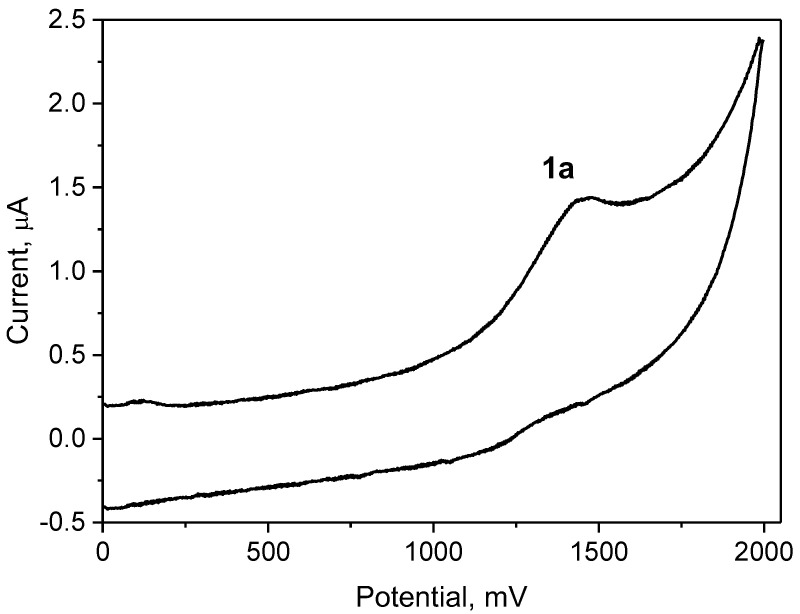
Cyclic voltammperogram of compound 1a in anhydrous acetonitrile. Solution contained tetra-*n*-butylammonium perchlorate as the supporting electrolyte and was deoxygenated prior to analysis by purging with helium for 15 min. Scan rate was 100 mVs^−1^.

To reveal the substituent effect on an oxidation potential, the cyclic voltammograms of the aminocinnamic aldehydes were recorded in anhydrous acetonitrile as illustrated in Figure 3. The results of the electrochemical measurements for all compounds are collected in Table 2.

In all cases, the electrochemical oxidation of the aminocinnamic aldehydes is irreversible. The location of the oxidation peaks depends on the alkylamino substituent. Compared to the parent compound (1a), the aminocinnamic aldehydes are more easily oxidized. In the case of 1j containing the stiffened amine group, the oxidation proceeds at the lowest potential. Only compound 1g, which is a morpholine derivative, has a lower tendency to lose electrons.

## 3. Experimental Section

### 3.1. Materials and Methods

Starting reagents and solvents were purchased from Aldrich Chemical Co. Aldehydes were purified using Büchi Sepacore^®^ Chromatography system (C-605, C-610) with a UV-Vis detector (C-640). Melting points were determined on the Büchi melting point apparatus MP-1. The ^1^H (400 MHz) and ^13^C (100 MHz) NMR spectra were recorded on a Bruker Ascent^TM^ 400 NMR spectrometer. Dimethylsulfoxide (DMSO-*d_6_*) was used as the solvent and tetramethylsilane as the internal standard. The IR spectra were recorded using a Bruker spectrophotometer Vector 22 in the range of 400–4500 cm^−1^ by the KBr pellet technique. Elemental analysis was carried out by an Elementar Vario MACRO apparatus. The UV-Vis absorption spectra were recorded on a Shimadzu UV-Vis Multispec-1501 spectrophotometer. The fluorescence spectra were obtained with a Hitachi F-7100 spectrofluorometer. The fluorescence quantum yield (FQY; ϕ) was calculated according to Equation (1) using coumarin I in ethanol as a reference [27].

(1) $$\phi_{s}=\phi_{ref}\frac{I_{s}A_{ref}}{I_{ref}A_{s}}\cdot \frac{n_{s}^{2}}{n_{ref}^{2}}$$

where: *I* is the integrated intensity (area) (in units of photons); *n* is the refractive index. The absorbances (A) of both the sample (*s*) and reference (*ref*) solution at an excitation wavelength (370 nm) was ca. 0.1.

The oxidation potential (E_ox_) was measured by cyclic voltammetry using an Electrochemical Analyzer EA9C, MTM Cracow in anhydrous acetonitrile with 0.1 M of tetrabutylammonium perchlorate as the supporting electrolyte. The scan rate was 100 mV/s. The measurements were made in a typical three-electrode setup containing a platinum 1 mm electrode as the working electrode and platinum and Ag/AgCl as auxiliary and reference electrodes, respectively.

### 3.2. Synthesis

#### 3.2.1. Method A

An appropriate *p*-aminobenzaldehyde (40 mmol) was dissolved in 30 mL of concentrated sulfuric acid under vigorous stirring. Then, the solution was cooled to 0 °C, and 120 mmol of acetaldehyde was added dropwise within 3 h. After another hour, the reaction mixture was poured onto ice and neutralized with 20% NaOH. The precipitate was filtered, dried, and recrystallized from ethanol. Only the aldehydes 1h, 1i and 1j needed to be purified by flash chromatography on silica gel (pore size 60 Å, 230–400 mesh particle size) using chloroform as the mobile phase [23].

#### 3.2.2. Method B

Anhydrous chloroform (20 mL), an appropriate aromatic amine (65 mmol) and 3-dimethylaminoacrolein (55 mmol) were placed in a flask equipped with a thermometer and a dropping funnel. The mixture was cooled to −10 °C, and a solution of 55 mmol of POCl_3_ in 8 mL of chloroform was added, keeping the temperature below 0 °C. Then, the mixture was allowed to warm up to room temperature and stirred overnight. It was next heated to 60 °C and kept at this temperature for 2 h. Chloroform was distilled off under reduced pressure, and 25 mL of methanol was added with stirring and cooling. The reaction mixture was poured onto 50 g of crushed ice and neutralized with 20% NaOH. The crude product was filtered and recrystallized from ethanol. As before, the aldehydes 1h, 1i and 1j were purified by flash chromatography on silica gel (pore size 60 Å, 230–400 mesh particle size) using chloroform as the mobile phase [25].

3-[4-(Dimethylamino)phenyl]prop-2-enal (1a)

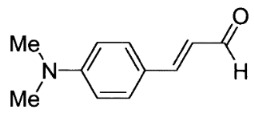

The compound was obtained as a brown solid; yield: 54% (method A), 61% (method B); mp 140 °C; ^1^H NMR (400 MHz, DMSO-*d*_6_): δ 9.53 (d, *J* = 8.0 Hz, 1H, CHO), 7.59–7.55 (m, 3H), 6.75 (d, *J* = 9.0 Hz, 2H), 6.59 (dd, *J* = 8.0 Hz, *J* = 15.8 Hz, 1H), 3.01 (s, 6H, N(CH_3_)_2_); ^13^C NMR (100 MHz, DMSO-*d*_6_): δ 193.9 (CHO), 154.7 (CH), 152.7 (C), 131.1 (CH), 123.7 (CH), 121.8 (C), 112.2 (CH), 40.1 (N(CH_3_)_2_); IR (KBr): 1664, 1600, 1529, 1373, 1140, 973, 811.

Anal. calculated for C_11_H_13_NO: C, 75.40; H, 7.48; N, 7.99. Found: C, 75.51; H, 7.40; N, 7.94.

3-[4-(Diethylamino)phenyl]prop-2-enal (1b)

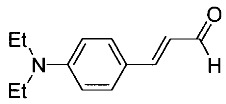

The compound was obtained as a brown solid; yield: 39% (method A), 55% (method B); mp 73 °C; ^1^H NMR (400 MHz, DMSO-*d*_6_): δ 9.52 (d, *J* = 8.0 Hz, 1H, CHO), 7.56–7.52 (m, 3H), 6.71 (d, *J* = 9.0 Hz, 2H), 6.55 (dd, *J* = 8.0 Hz, *J* = 15.9 Hz, 1H), 3.42 (q, *J* = 6.9 Hz, 4H, NCH_2_), 1.12 (t, *J* = 7.0 Hz, 6H, CH_3_); ^13^C NMR (100 MHz, DMSO-*d*_6_): δ 193.9 (CHO), 154.7 (CH), 150.3 (C), 131.5 (CH), 123.2 (CH), 121.0 (C), 111.6 (CH), 44.4 (NCH_2_), 12.9 (CH_3_); IR (KBr): 1649, 1596, 1525, 1415, 1187, 1135, 813.

Anal. calculated for C_13_H_17_NO: C, 76.81; H, 8.43; N, 6.89. Found: C, 76.90; H, 8.35; N, 6.86.

3-[4-(Dimethylamino)-2-methylphenyl]prop-2-enal (1c)

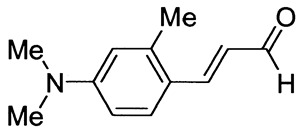

The compound was obtained as a dark red solid; yield: 49% (method A), 58% (method B); mp 110 °C; ^1^H NMR (400 MHz, DMSO-*d*_6_): δ 9.57 (d, *J* = 8.0 Hz, 1H, CHO), 7.80 (d, *J* = 15.8 Hz, 1H, -CH=) 7.65 (d, *J* = 9.2 Hz, 1H), 6.61-6.48 (m, 3H), 2.99 (s, 6H, N(CH_3_)_2_), 2.40 (s, 3H, ArCH_3_); ^13^C NMR (100 MHz, DMSO-*d*_6_): δ 193.7 (CHO), 152.0 (C), 151.0 (CH), 139.9 (C), 128.6 (CH), 123.6 (CH), 119.8 (C), 113.0 (CH), 110.0 (CH), 39.6 (N(CH_3_)_2_), 19.9 (ArCH_3_); IR (KBr): 1650, 1597, 1580, 1374, 1296, 1212, 1154, 1099, 811.

Anal. calculated for C_12_H_15_NO: C, 76.16; H, 7.99; N, 7.40. Found: C, 76.23; H, 7.91; N, 7.38.

3-[4-(Dimethylamino)-2,6-dimethylphenyl]prop-2-enal (1d)

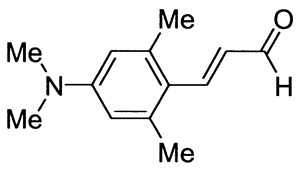

The compound was obtained as a dark yellow solid; yield: 0% (method A), 36% (method B); mp 111 °C; ^1^H NMR (400 MHz, DMSO-*d*_6_): δ 9.55 (d, *J* = 7.7 Hz, 1H, CHO), 7.81 (d, *J* = 16 Hz, 1H, -CH=), 6.50 (s, 2H), 6.31 (dd, *J* = 7.7 Hz, *J* = 16.0 Hz, 1H), 2.97 (s, 6H, N(CH_3_)_2_), 2.38 (s, 6H, ArCH_3_); ^13^C NMR (100 MHz, DMSO-*d*_6_): δ 195.2 (CHO), 152.0 (CH), 151.4 (C), 140.6 (C), 128.8 (CH), 120.2 (C), 112.7 (CH), 40.0 (N(CH_3_)_2_), 22.8 (ArCH_3_); IR (KBr): 1656, 1583, 1510, 1366, 1146, 827.

Anal. calculated for C_13_H_17_NO: C, 76.81; H, 8.43; N, 6.89. Found: C, 76.93; H, 8.41; N, 6.88.

3-[4-(Pyrrolidin-1-yl)phenyl]prop-2-enal (1e)

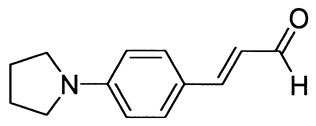

The compound was obtained as a brown solid; yield: 45% (method A), 54% (method B); mp 131 °C; ^1^H NMR (400 MHz, DMSO-*d*_6_): δ 9.52 (d, *J* = 8.0 Hz, 1H, CHO), 7.57–7.54 (m, 3H), 6.60-6.54 (m, 3H), 3.32 (t, *J* = 4.5 Hz, 4H, NCH_2_), 1.97 (m, 4H, CH_2_); ^13^C NMR (100 MHz, DMSO-*d*_6_): δ 193.8 (CHO), 155.0 (CH), 150.2 (C), 131.3 (CH), 123.2 (CH), 121.3 (C), 112.2 (CH), 47.7 (NCH_2_), 25.4 (CH_2_); IR (KBr): 1667, 1594, 1526, 1398, 1187, 1136, 971, 810.

Anal. calculated for C_13_H_15_NO: C, 77.58; H, 7.51; N, 6.96. Found: C, 77.61; H, 7.43; N, 6.98.

3-[4-(Piperidin-1-yl)phenyl]prop-2-enal (1f)

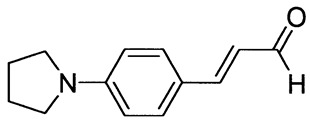

The compound was obtained as a dark brown solid; yield: 52% (method A), 60% (method B); mp 87 °C; ^1^H NMR (400 MHz, DMSO-*d*_6_): δ 9.55 (d, *J* = 7.9 Hz, 1H, CHO), 7.59–7.55 (m, 3H), 6.96 (d, *J* = 9.0 Hz, 2H), 6.61 (dd, *J* = 8.0 Hz, *J* = 15.8 Hz, 1H), 3.33 (t, *J* = 8.7 Hz, 4H, NCH_2_), 1.58 (m, 6H); ^13^C NMR (100 MHz, DMSO-*d*_6_): δ 194.1 (CHO), 154.3 (CH), 153.3 (C), 131.1 (CH), 124.5 (CH), 123.2 (C), 114.5 (CH), 48.4 (NCH_2_), 25.3 (CH_2_), 24.4 (CH_2_); IR (KBr): 1653, 1600, 1516, 1243, 1137, 1124, 811.

Anal. calculated for C_14_H_17_NO: C, 78.11; H, 7.96; N, 6.51. Found: C, 78.19; H, 7.88; N, 6.45.

3-[4-(Morpholin-4-yl)phenyl]prop-2-enal (1g)

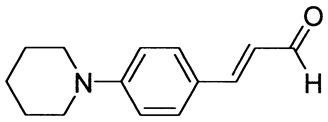

The compound was obtained as a dark brown solid; yield: 52% (method A), 58% (method B); mp 109 °C; ^1^H NMR (400 MHz, DMSO-*d*_6_): δ 9.57 (d, *J* = 7.9 Hz, 1H, CHO), 7.62–7.58 (m, 3H), 6.99 (d, *J* = 9.0 Hz, 2H), 6.66 (dd, *J* = 7.9 Hz, *J* = 15.7 Hz, 1H), 3.73 (t, *J* = 4.9 Hz, 4H, OCH_2_), 3.26 (t, *J* = 4.9 Hz, 4H, NCH_2_); ^13^C NMR (100 MHz, DMSO-*d*_6_): δ 194.3 (CHO), 154.1 (CH), 153.4 (C), 130.9 (CH), 125.2 (CH), 124.5 (C), 114.5 (CH), 66.2 (OCH_2_), 47.4 (NCH_2_); IR (KBr): 1659, 1601, 1518, 1234, 1137, 1123, 927, 811, 629.

Anal. calculated for C_13_H_15_NO_2_: C, 71.87; H, 6.96; N, 6.45. Found: C, 71.93; H, 6.92; N, 6.41.

3-(1-Methyl-2,3-dihydro-1H-indol-5-yl)prop-2-enal (1h)

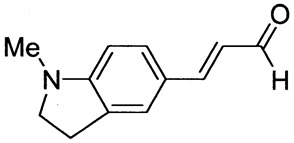

Purified by flash chromatography (silica gel, chloroform as eluent). The compound was obtained as a yellow solid; yield: 37% (method A), 52% (method B); mp 65 °C; ^1^H NMR (400 MHz, DMSO-*d*_6_): δ 9.50 (d, *J* = 7.9 Hz, 1H, CHO), 7.53 (d, *J* = 15.6 Hz, 1H, -CH=), 7.43 (s, 1H), 7.38 (d, *J* = 8.2 Hz, 1H), 6.54 (dd, *J* = 8.0 Hz, *J*=15.8 Hz, 1H), 6.51 (d, *J* = 7.8 Hz, 1H), 3.45 (t, *J* = 8.4 Hz, 2H, NCH_2_), 2.95 (t, *J* = 8.3 Hz, 2H), 2.81 (s, 3H, NCH_3_); ^13^C NMR (100 MHz, DMSO-*d*_6_): δ 193.9 (CHO), 156.4 (C), 155.1 (CH), 132.0 (CH), 131.2 (C), 124.4 (CH), 123.2 (CH), 123.2 (C), 106.0 (CH), 55.0 (NCH_2_), 34.6 (NCH_3_), 27.7 (ArCH_2_); IR (KBr): 1657, 1595, 1505, 1329, 1301, 1130, 1081, 813.

Anal. calculated for C_12_H_13_NO: C, 76.98; H, 7.00; N, 7.48. Found: C, 77.04; H, 7.08; N, 7.41.

3-(1-Methyl-1,2,3,4-tetrahydroquinolin-6-yl)prop-2-enal (1i)

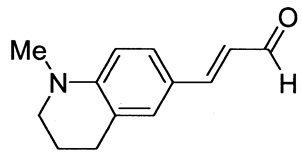

Purified by flash chromatography (silica gel, chloroform as eluent). The compound was obtained as a brown solid; yield: 43% (method A), 55% (method B); mp 65 °C; ^1^H NMR (400 MHz, DMSO-*d*_6_): δ 9.50 (d, *J* = 8.0 Hz, 1H, CHO), 7.49 (d, *J* = 15.6 Hz, 1H, -CH=), 7.37 (d, *J* = 8.5 Hz, 1H), 7.29, (s, 1H), 6.59 (d, *J* = 8.6 Hz, 1H), 6.53 (dd, *J* = 8.0 Hz, *J* = 15.5 Hz, 1H), 3.32 (t, *J* = 5.7 Hz, 2H, NCH_2_), 2.94 (s, 3H, NCH_3_), 2.71 (t, *J* = 6.2 Hz, 2H, ArCH_2_), 1.88 (m, 2H); ^13^C NMR (100 MHz, DMSO-*d*_6_): δ 193.8 (CHO), 154.9 (CH), 149.4 (C), 130.1 (CH), 129.5 (CH), 123.1 (CH), 122.6 (C), 121.5 (C), 110.6 (CH), 50.9 (NCH_2_), 38.8 (NCH_3_), 27.6 (ArCH_2_), 21.7 (CH_2_); IR (KBr): 1664, 1595, 1525, 1322, 1205, 1137, 807.

Anal. calculated for C_13_H_15_NO: C, 77.58; H, 7.51; N, 6.96. Found: C, 77.52; H, 7.43; N, 6.91.

3-(9-Julolidyl)prop-2-enal (1j)

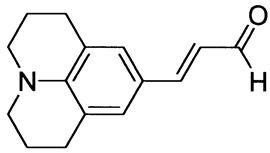

Purified by flash chromatography (silica gel, chloroform as eluent). The compound was obtained as a brown solid; yield: 32% (method A), 47% (method B); mp 125 °C; ^1^H NMR (400 MHz, DMSO-*d*_6_): δ 9.47 (d, *J* = 8.0 Hz, 1H, CHO), 7.42 (d, *J* = 15.5 Hz, 1H, -CH=), 7.11 (s, 1H), 6.47 (dd, *J* = 8.0 Hz, *J* = 15.5 Hz, 1H), 3.24 (t, *J* = 5.8 Hz, 4H, NCH_2_), 2.68 (t, *J* = 6.3 Hz, 4H, ArCH_2_), 1.86 (m, 4H, CH_2_); ^13^C NMR (100 MHz, DMSO-*d*_6_): δ 193.6 (CHO), 155.1 (CH), 145.9 (C), 128.6 (CH), 122.6 (CH), 121.0 (C), 120.6 (C), 49.7 (NCH_2_), 27.5 (CH_2_), 21.4 (CH_2_); IR (KBr): 1654, 1588, 1523, 1319, 1128, 969.

Anal. calculated for C_15_H_17_NO: C, 79.26; H, 7.54; N, 6.16. Found: C, 79.21; H, 7.50; N, 6.19.

## 4. Conclusions

*p*-Aminocinnamic aldehydes were prepared from the reaction of either acetaldehyde with aromatic aldehydes bearing appropriate substituents (Method A) or aromatic amine and 3-dimethylaminoacrolein in the presence of POCl_3_ (Method B). The desired products were obtained at a low to moderate yield with slightly higher values of ca. 6–16% achieved using Method B. The yields of the reactions carried out according to method A are 32–54%, while for Method B, they are in the range of 36–61%. In addition to the better yield, an important advantage of Method B is the use of relatively readily available aromatic amines instead of the often difficult to obtain benzaldehydes. Moreover, the 4-(N,N-dimethylamino)-2,6-dimethylcinnamaldehyde (1d), probably due to spatial hindrance, could only be synthesized by Method B. It should also be noted that six of the synthesized aldehydes (1c, 1d, 1e, 1f, 1h, 1i) have not been described in the literature so far.

Spectroscopic analysis showed that the absorption and fluorescence maxima of the cinnamic aldehydes, due to the additional double bond, are redshifted by approximately 45 and 75 nm, respectively, relative to the corresponding benzaldehydes. Moreover, their spectroscopic properties depend on the amine substituent present in the phenyl ring and solvent used. The structure of the cinnamaldehydes also influences the location of the oxidation peaks.

## Figures and Tables

**Table 1:** Absorption maxima wavelength (λ_max abs_; nm), molar absorption coefficient (ε_max_; M^−1^ cm^−1^), fluorescence maxima wavelength (λ_max fl_; nm), fluorescence quantum yield (ϕ_fl_; %) and Stokes shift (∆ν^St^; cm^−1^) in ethyl acetate and methanol.

Abbr.	Ethyl Acetate			Methanol		
	λ_max abs_ (ε_max_)	λ_max fl_ (ϕ_fl_)	∆ν^St^	λ_max abs_ (ε_max_)	λ_max fl_ (ϕ_fl_)	∆ν^St^
1a	372.5 (43,000)	437 (0.42)	3962	389 (37,700)	474 (0.33)	4610
1b	379.5 (44,500)	438 (0.69)	3519	395.5 (45,700)	477 (0.20)	4320
1c	378 (35,500)	444 (0.33)	3933	395.5 (36,900)	474 (0.14)	4187
1d	374 (35,200)	474 (0.71)	5641	392 (34,900)	not fluorescent	-
1e	379.5 (43,900)	440 (0.61)	3623	396.5 (40,200)	476 (0.30)	4212
1f	368.5 (33,800)	440 (0.99)	4410	385 (31,500)	481 (0.28)	5184
1g	357 (31,400)	438 (0.51)	5180	368 (28,900)	486 (0.38)	6598
1h	378 (33,800)	460 (1.19)	4716	395.5 (33,900)	485 (0.18)	4666
1i	385 (40,500)	453 (1.14)	3899	402.5 (35,600)	486 (0.26)	4269
1j	396.5 (41,800)	460 (1.88)	3482	417.5 (38,300)	488 (0.24)	3460

**Table 2:** The measured oxidation potential of the tested aminocinnamaldehydes.

Abbr.	E_ox_ (mV)	Abbr.	E_ox_ (mV)
1a	1285	1f	1228
1b	1219	1g	1300
1c	1194	1h	1093
1d	1081	1i	1132
1e	1186	1j	870

## Data Availability

The data is provided by Marek Pietrzak (UTP University of Science and Technology), please contact marek@utp.edu.pl. The data is not publicly available apart from the data contained in the article or Supplementary Materials.

## Supplementary Materials

Supplementary data related to NMR, IR, UV-Vis and fluorescence spectroscopy data for all tested compounds can be found online.

## Sample Availability

Samples of the compounds are available from the author (M. Pietrzak).

## References

[1] Li X., Gao X., Shi W., Ma H. (2014). Design strategies for water-soluble small molecular chromogenic and fluorogenic probes. Chem. Rev..

[2] Jiang X., Marinado T., Gabrielsson E., Hagberg D.P., Sun L., Hagfeldt A. (2010). Structural modification of organic dyes for efficient coadsorbent-free dye-sensitized solar cells. J. Phys. Chem. C..

[3] Chen S.-L., Yang L.-N., Li Z.-S. (2013). How to design more efficient organic dyes for dye-sensitized solar cells? Adding more sp2-hybridized nitrogen in the triphenylamine donor. J. Power Sources..

[4] Lavis D.L., Raines R.T. (2008). Bright Ideas for Chemical Biology. ACS Chem. Biol..

[5] Costero A.M., Banuls M.J., Aurell M.J., Ochando L.E., Domenech A. (2005). Cation and anion fluorescent and electrochemical sensors derived from 4,4′-substituted biphenyl. Tetrahedron.

[6] Krawczyk P., Pietrzak M., Janek T., Jędrzejewska B., Cysewski P. (2016). Spectroscopic and nonlinear optical properties of new chalcone fluorescent probes for bioimaging applications: A theoretical and experimental study. J. Mol. Model..

[7] Cook W.D., Chen F. (2011). Enhanced photopolymerization of dimethacrylates with ketones, amines, and iodonium salts: The CQ system. J. Poym. Sci. Pol. Chem..

[8] Jędrzejewska B., Pietrzak M., Rafiński Z. (2011). Phenyltrialkylborates as co-initiators with cyanine dyes in visible light polymerization of acrylates. Polymer.

[9] Hrobarik P., Hrobarikova V., Sigmundova I., Zahradnik P., Fakis M., Polyzos I., Persephonis P. (2011). Benzothiazoles with tunable electron-withdrawing strength and reverse polarity: A route to triphenylamine-based chromophores with enhanced two-photon absorption. J. Org. Chem..

[10] Mueller K., Faeh C., Diederich F. (2007). Fluorine in pharmaceuticals: Looking beyond intuition. Science..

[11] Grabowski Z.R., Rotkiewicz K., Rettig W. (2003). Structural changes accompanying intramolecular electron transfer: Focus on twisted intramolecular charge-transfer states and structures. Chem. Rev..

[12] Zakrzewska A., Gawinecki R., Kolehmainen E., Ośmiałowski B. (2005). ^13^C-NMR based evaluation of the electronic and steric interactions in aromatic amines. Int. J. Mol. Sci..

[13] Pietrzak M., Bajorek A. (2013). 5-Phenyl-1,2,3,4-tetrahydronaphthalene derivatives: Synthesis, spectroscopic and electrochemical investigation. Dyes Pigments.

[14] Heck R.F., Nolley J.P. (1972). Palladium-catalyzed vinylic hydrogen substitution reactions with aryl, benzyl, and styryl halides. J. Org. Chem..

[15] Pietrzak M., Jędrzejewska B., Mądrzejewska D., Bajorek A. (2017). Convenient synthesis of *p*-aminobenzoic acids and their methyl esters. Org. Prep. Proced. Int..

[16] Gadigennavar S., Ranganathan M., Sankararaman S. (2020). Which isomer is it, 1,2,5,6- or 1,4,5,8-tetrasubstituted cycloocta-1,3,5,7-tetraene? Synthesis of symmetrically tetrasubstituted cycloocta-1,3,5,7-tetraene derivatives. Org. Biomol. Chem..

[17] Wan J., Fan B., Thang S.H. (2021). Sonochemical preparation of polymer-metal nanocomposites with catalytic and plasmonic properties. Nanoscale Adv..

[18] Liu Q., Wang X., Yan H., Wu Y., Li Z., Gong S., Liu P., Liu Z. (2015). Benzothiazole-enamide-based BF2 complexes: Luminophores exhibiting aggregation-induced emission, tunable emission and highly efficient solid-state emission. J. Mater. Chem. C.

[19] Ośmiałowski B., Petrusevich E.F., Antoniak M.A., Grela I., Bin Jassar M.A., Nyk M., Luis J.M., Jędrzejewska B., Zaleśny R., Jacquemin D. (2020). Controlling two-photon action cross section by changing a single heteroatom position in fluorescent dyes. J. Phys. Chem. Lett..

[20] Jędrzejewska B., Krawczyk P., Pietrzak M., Gordel M., Matczyszyn K., Samoć M., Cysewski P. (2013). Styryl dye possessing donor-π-acceptor structure-synthesis, spectroscopic and computational studies. Dyes Pigments.

[21] Matsumura K., Ono M., Yoshimura M., Kimura H., Watanabe H., Okamoto Y., Ihara M., Takahashi R., Saji H. (2013). Synthesis and biological evaluation of novel styryl benzimidazole derivatives as probes for imaging of neurofibrillary tangles in Alzheimer’s disease. Bioorg. Med. Chem..

[22] Shi P.C., Jiang X.D., Gao R.N., Dou Y.Y., Zhao W.L. (2015). Synthesis and application of Vis/NIR dialkylaminophenylbuta-1,3-dienyl borondipyrromethene dyes. Chin. Chem. Lett..

[23] Wang N., Wang C. (1971). The Effect of Para Substitution on the Rate of Alkaline Hydrolysis of Ethyl 5-Ethyl-2,4-pentadienoates. J. Org. Chem..

[24] Togninelli A., Gevariya H., Alongi M., Botta M. (2007). An improved general method for palladium catalyzed alkenylations and alkynylations of aryl halides under microwave conditions. Tetrahedron Lett..

[25] Ullrich F.W., Breitmaier E. (1983). Vinylogous vilsmeier formylation with 3-(N,N-dimethylamino)-acroleins. Synthesis.

[26] Szukalski A., Jędrzejewska B., Krawczyk P., Bajorek A. (2020). An optical modulator on the pyrazolone-BASED bi-component system. Dyes Pigments.

[27] Olmsted J. (1979). Calorimetric determinations of absolute fluorescence quantum yields. J. Phys. Chem..

