# Supplementary material for: Aromatic Amines in Organic Synthesis. Part II. p-Aminocinnamaldehydes
Source: Molecules. 2021 Jul 19;26(14):4360. doi: 10.3390/molecules26144360 (PMC8306840; doi:10.3390/molecules26144360)

SUPPORTING INFORMATION

Aromatic Amines in Organic Synthesis. Part II. *p*-Aminocinnamaldehydes.

*Marek Pietrzak** *and Beata Jędrzejewska*

Faculty of Chemical Technology and Engineering, UTP University of Sciences and Technology, Seminaryjna 3, 85-326 Bydgoszcz, Poland

	page
Electronic absorption spectra in methanol and ethyl acetate	**S2**
Fluorescence spectra in methanol and ethyl acetate	**S3**
^{1}H and ^{13}C NMR spectra	**S4-S13**
IR spectra	**S14-S23**

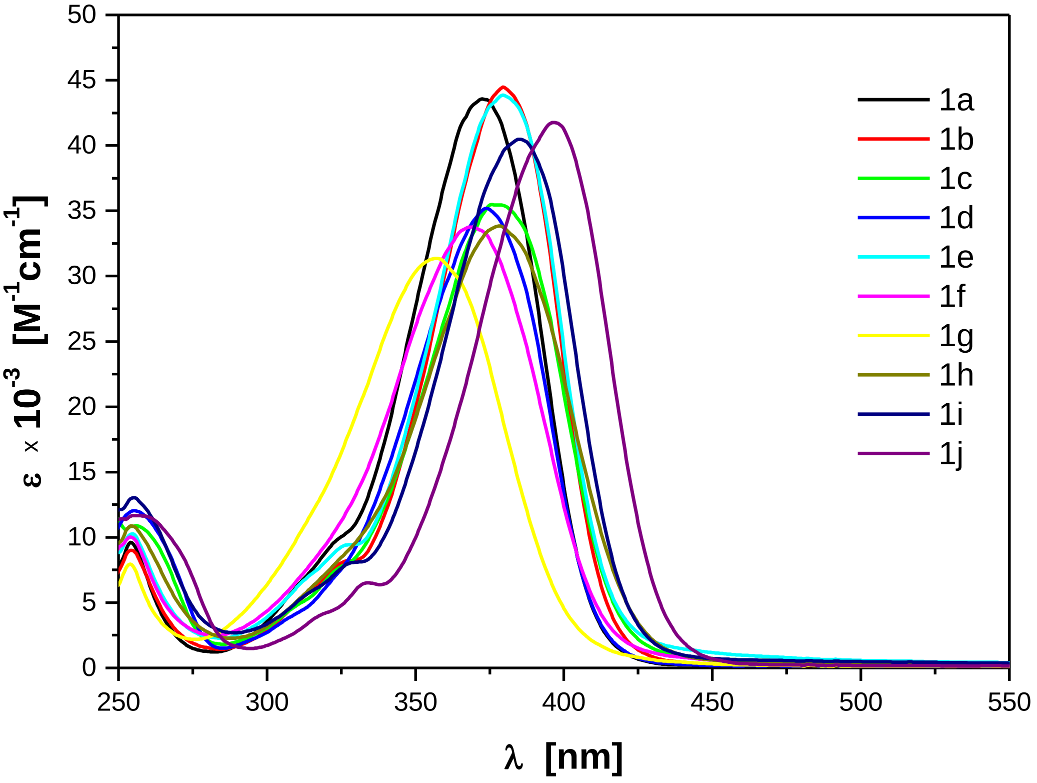

Fig. S1. Electronic absorption spectra of tested *p*-aminocinnamaldehydes in ethyl acetate.

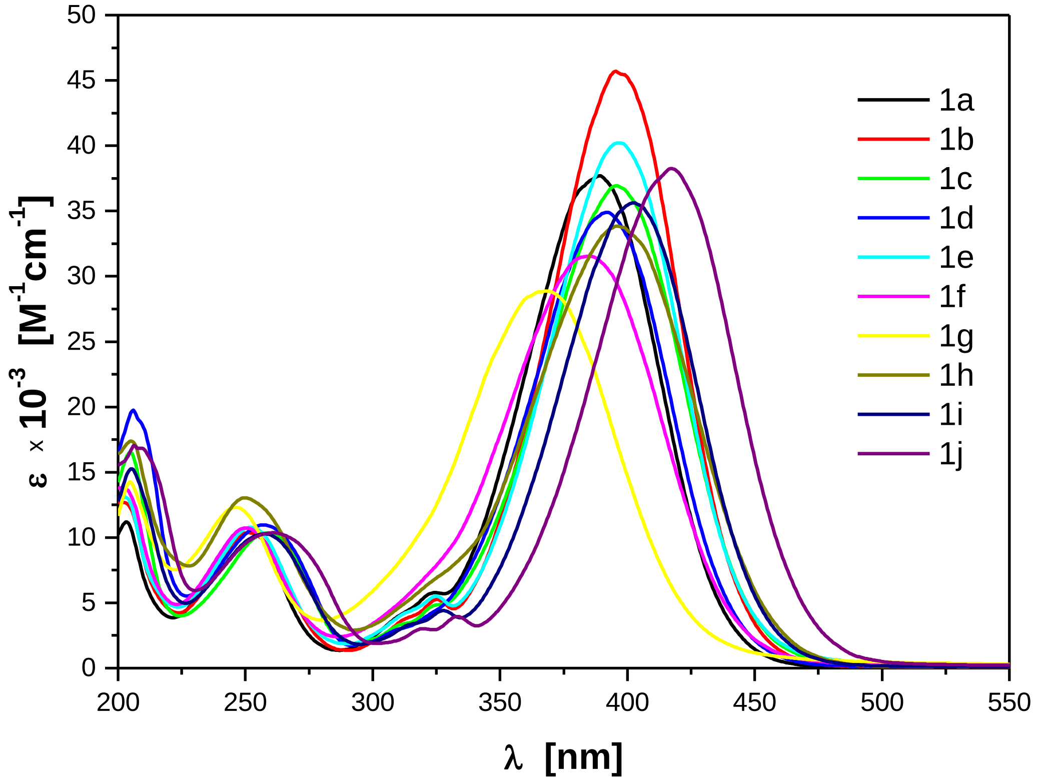

Fig. S2. Electronic absorption spectra of tested *p*-aminocinnamaldehydes in methanol.

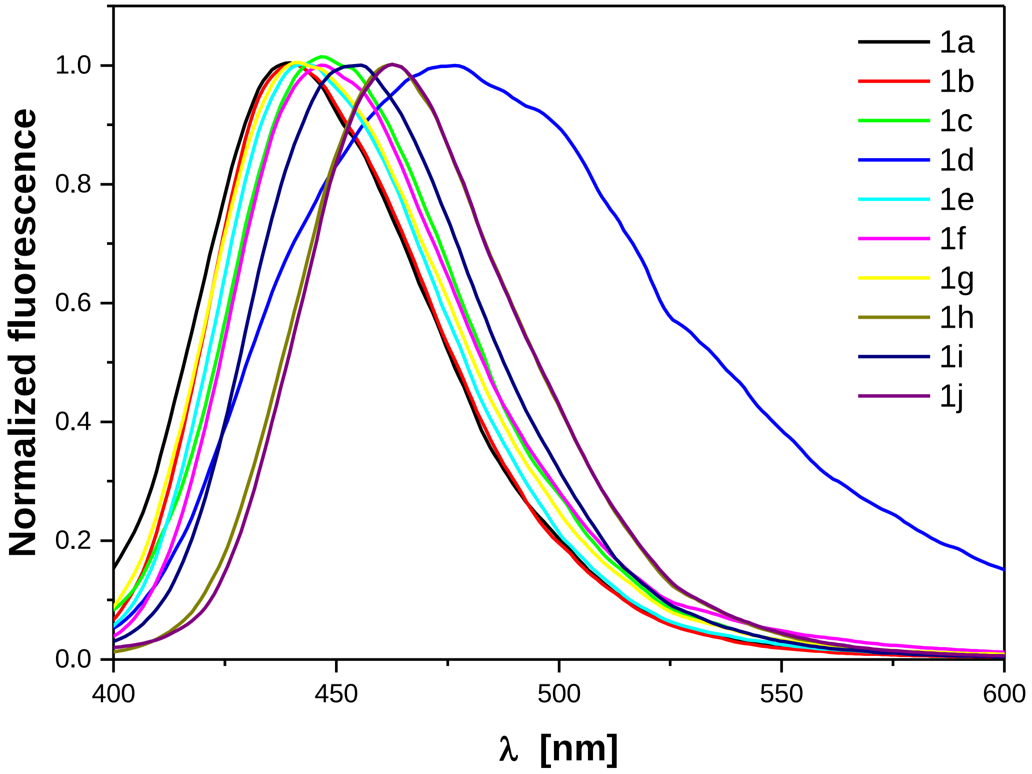

Fig. S3. Normalized fluorescence spectra of tested *p*-aminocinnamaldehydes in ethyl acetate.

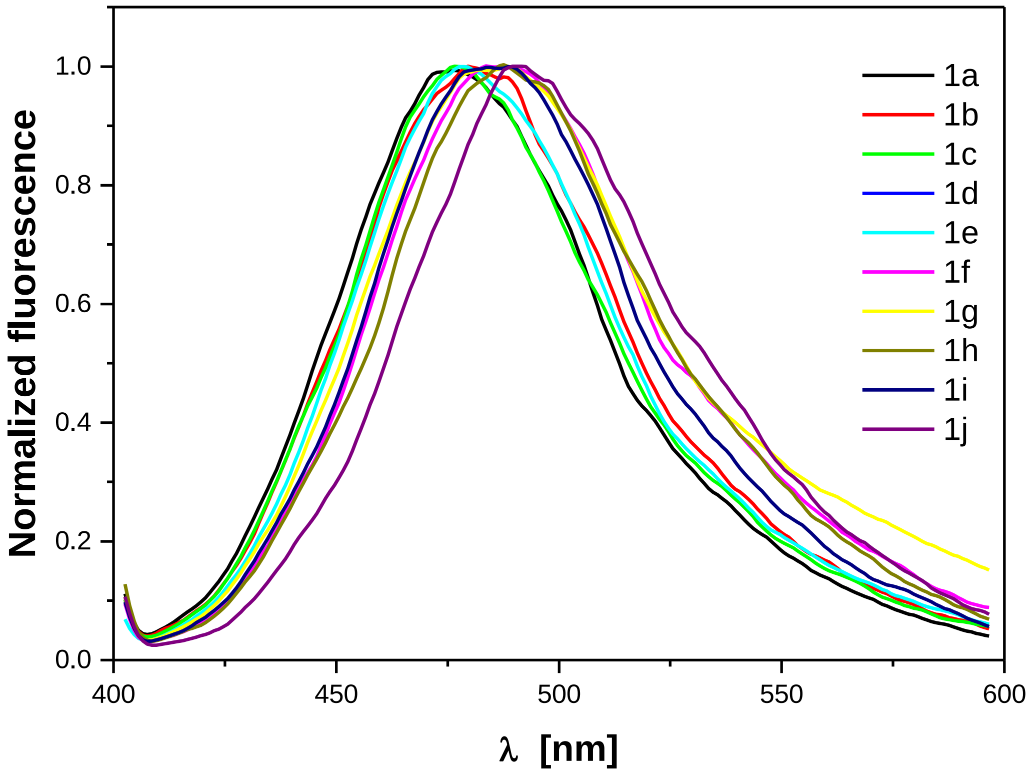

Fig. S4. Normalized fluorescence spectra of tested *p*-aminocinnamaldehydes in methanol.

^{1}H NMR spectrum of **1a**

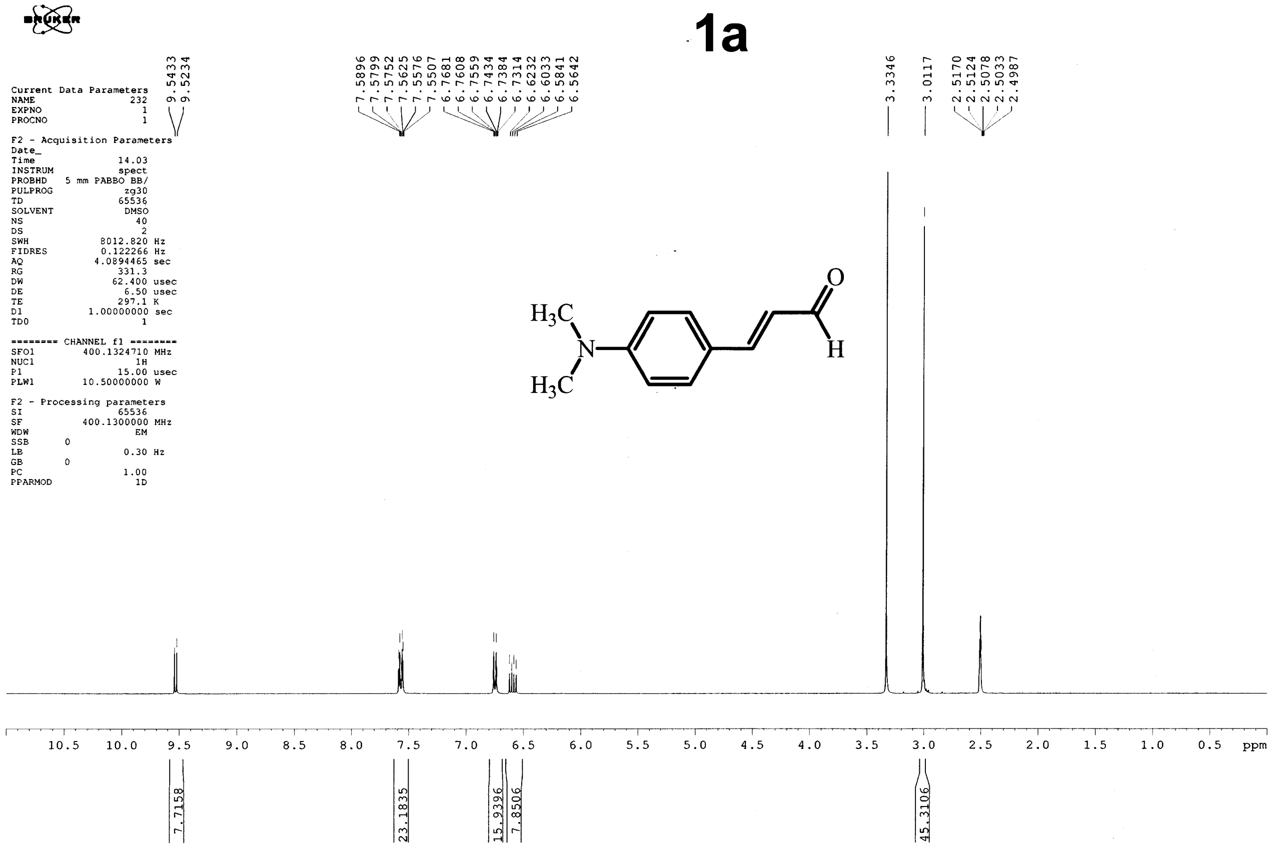

^{13}C NMR spectrum of **1a**

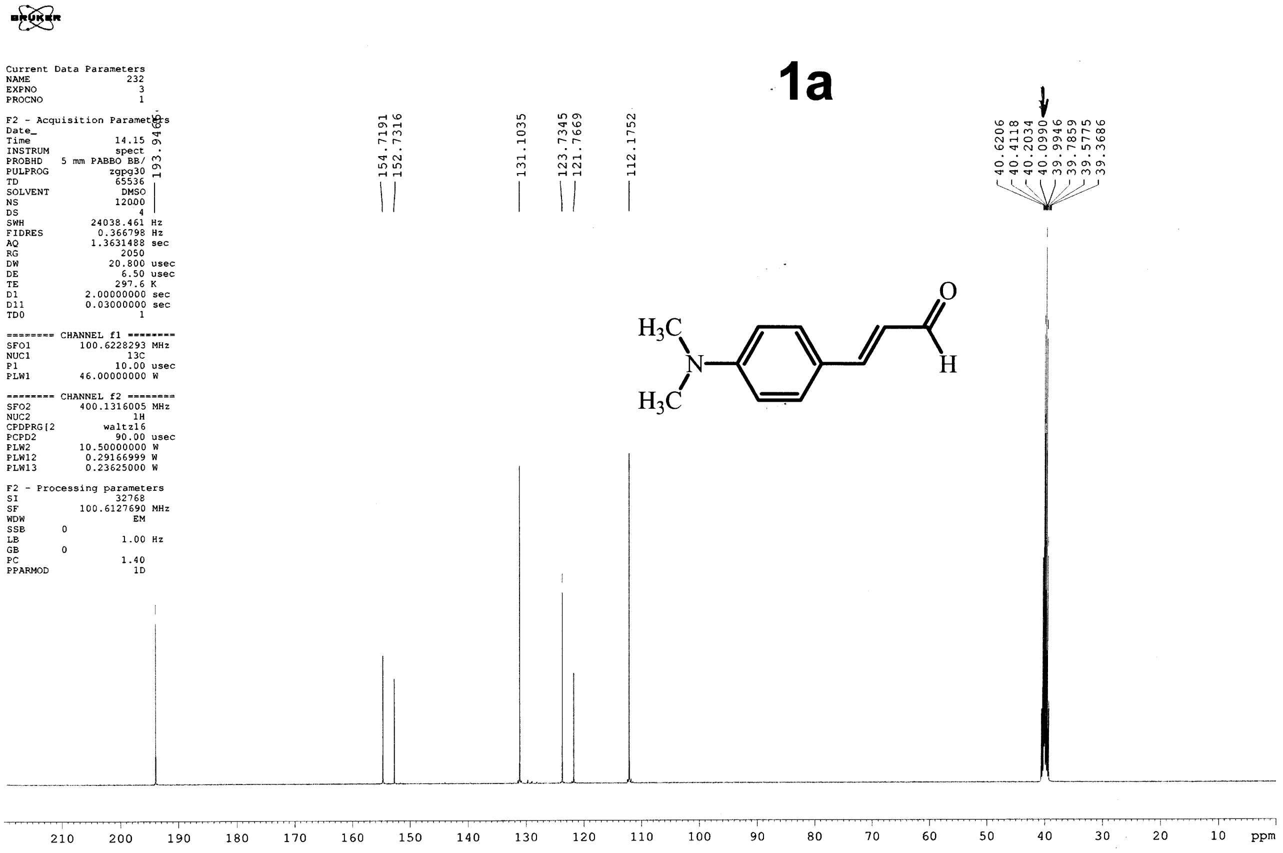

^{1}H NMR spectrum of **1b**

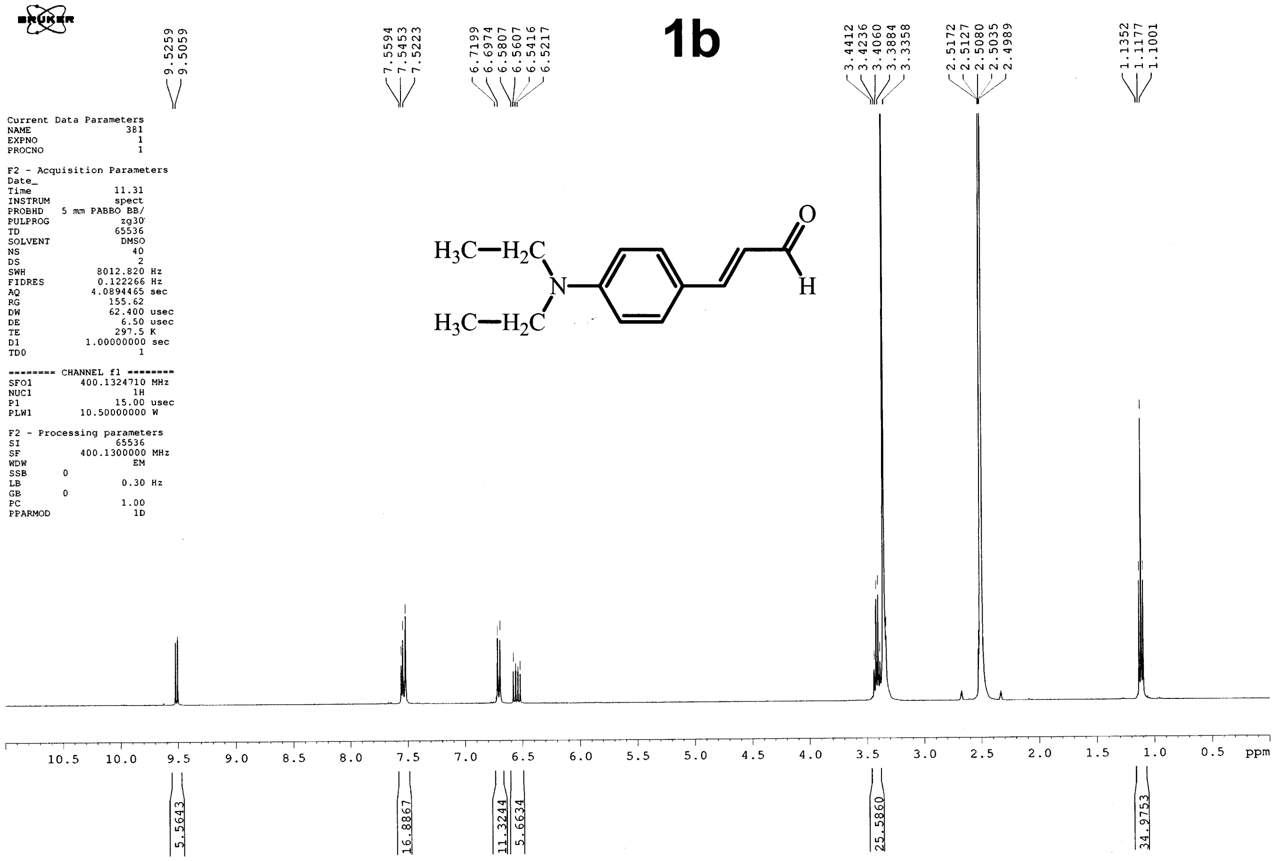

^{13}C NMR spectrum of **1b**

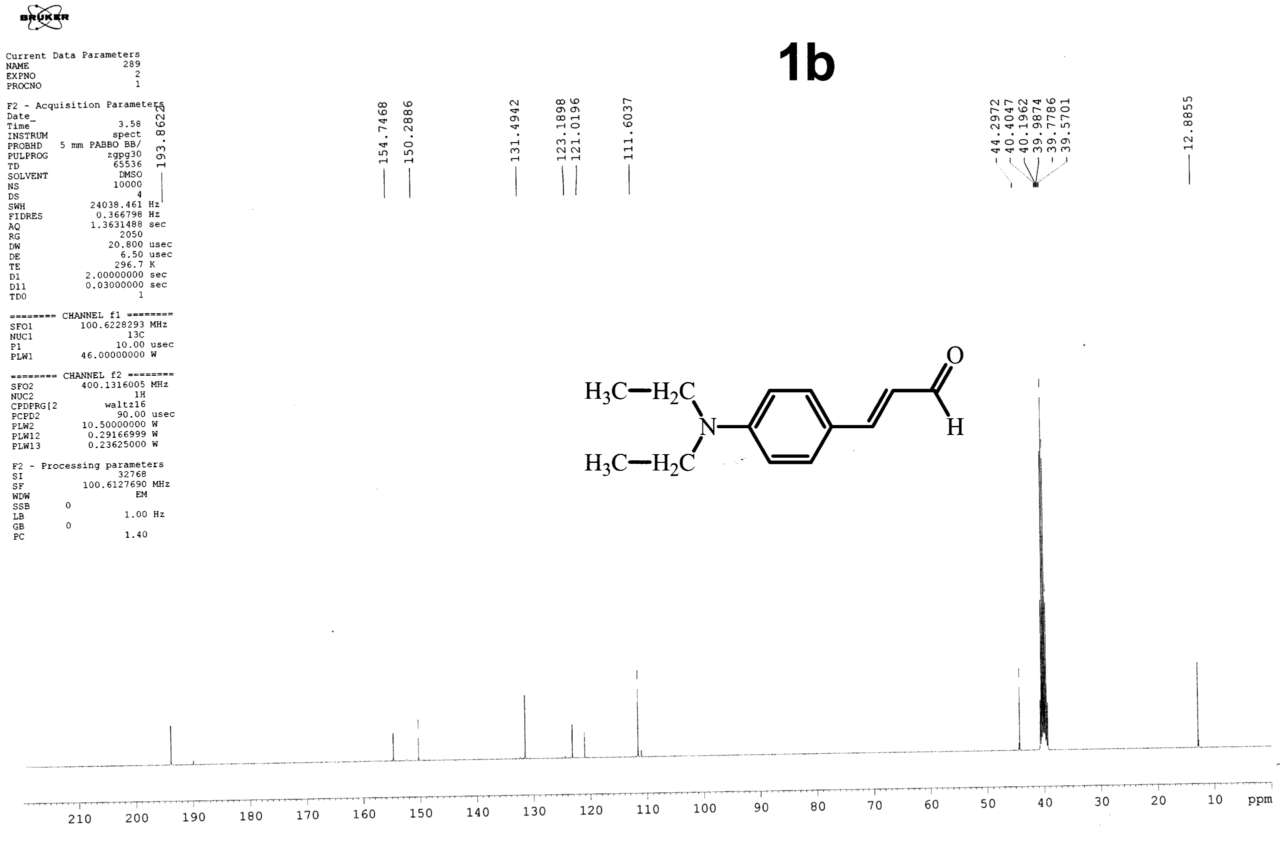

^{1}H NMR spectrum of **1c**

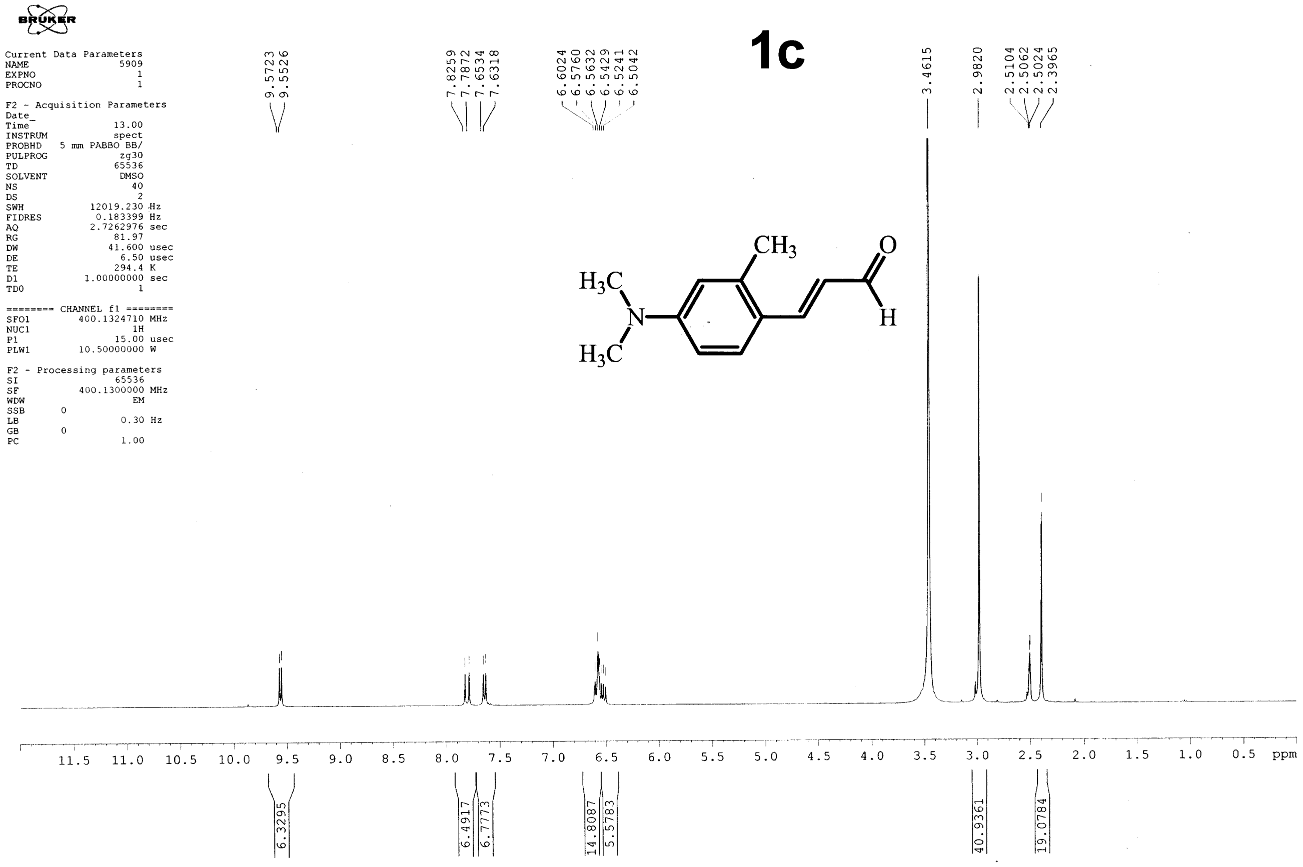

^{13}C NMR spectrum of **1c**

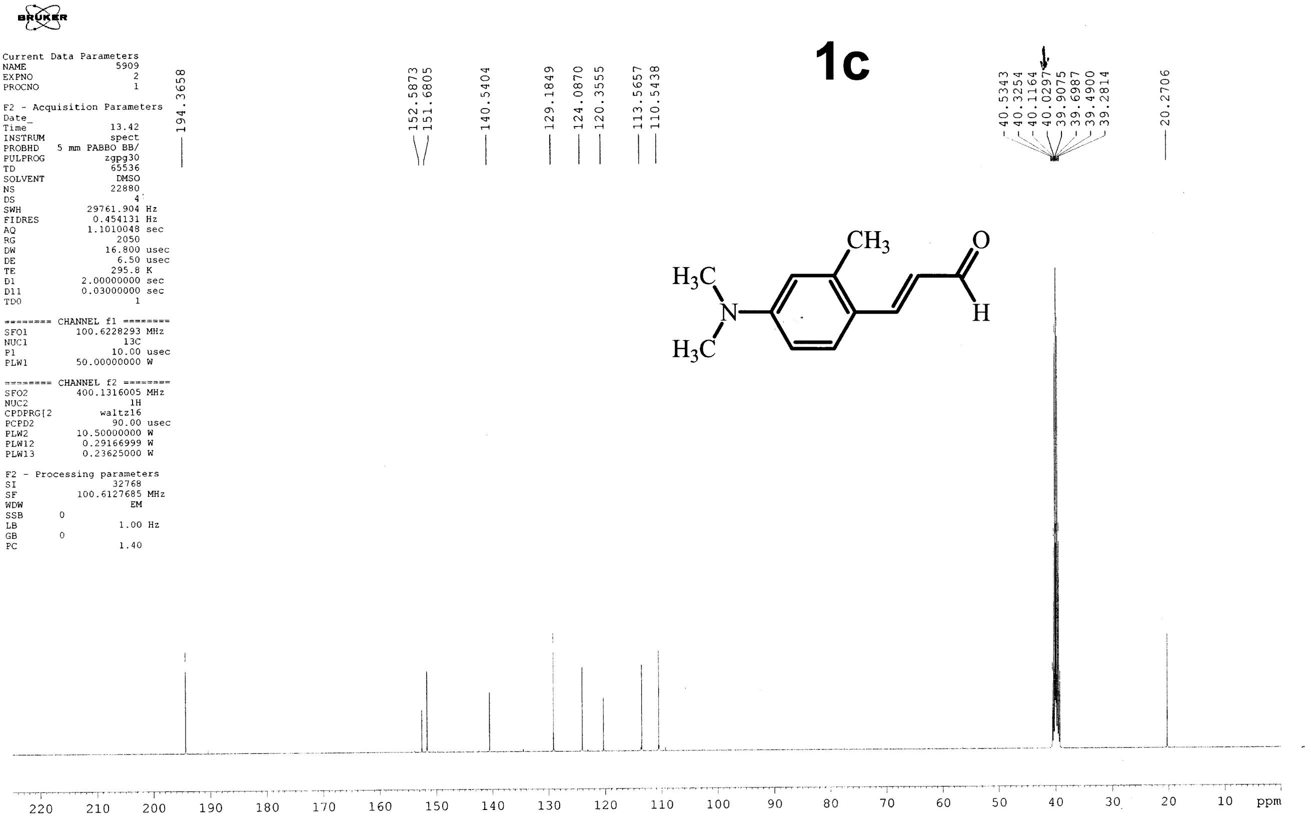

^{1}H NMR spectrum of **1d**

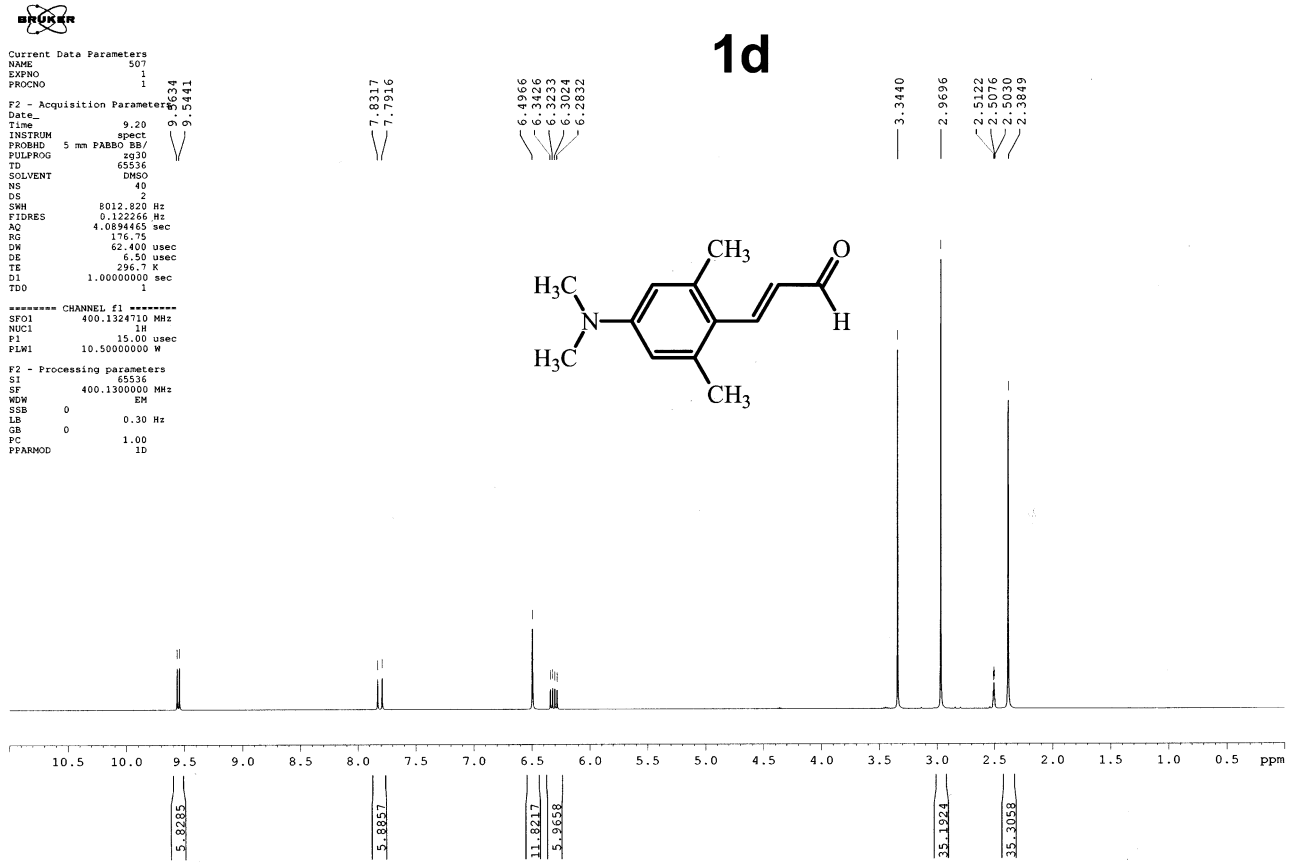

^{13}C NMR spectrum of **1d**

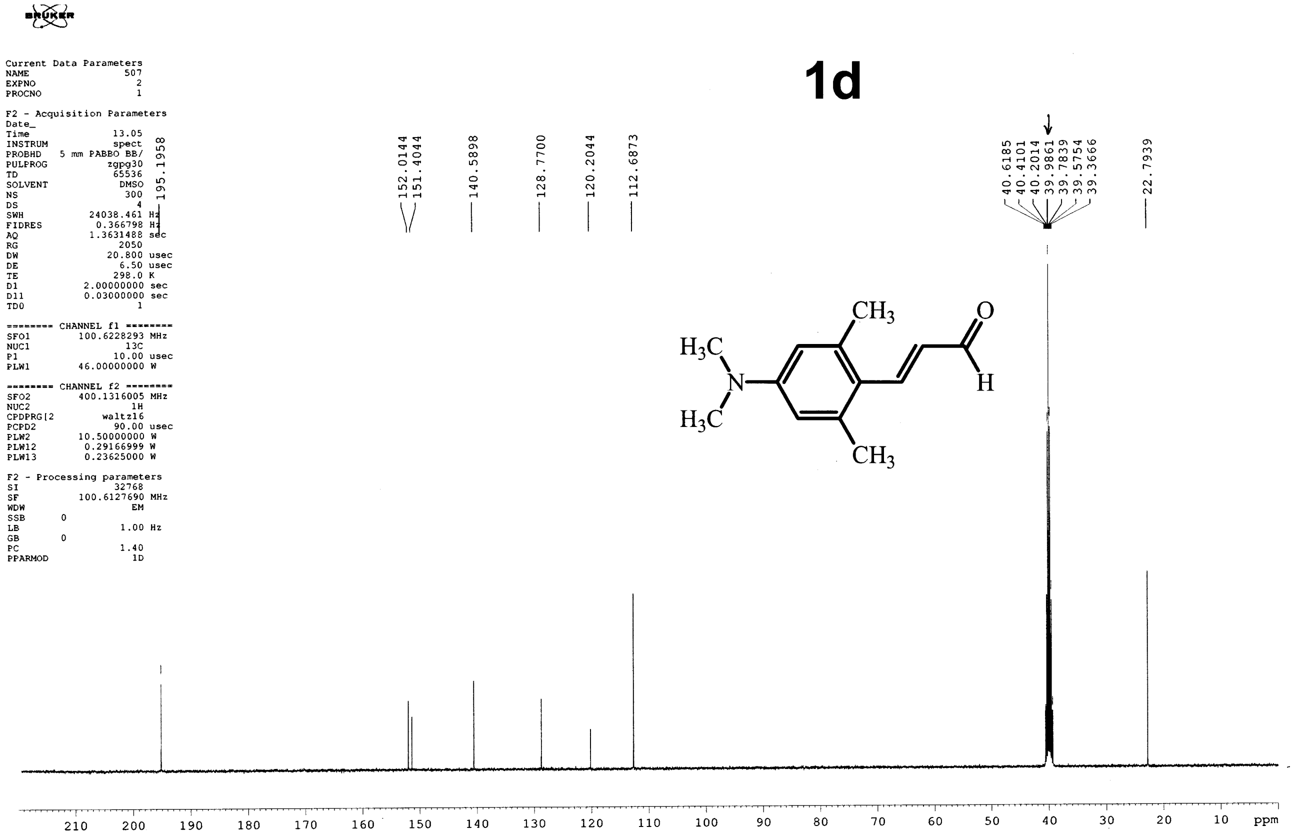

^{1}H NMR spectrum of **1e**

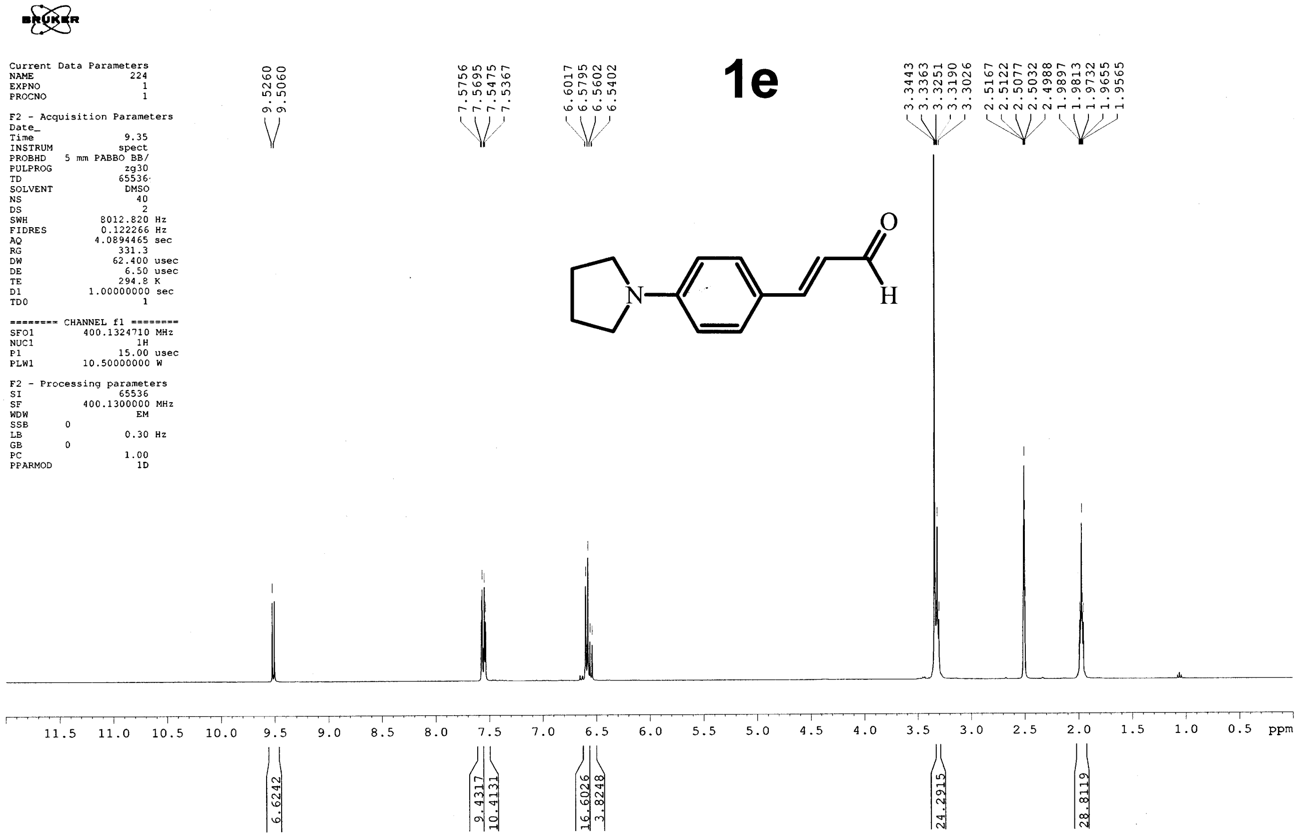

^{13}C NMR spectrum of **1e**

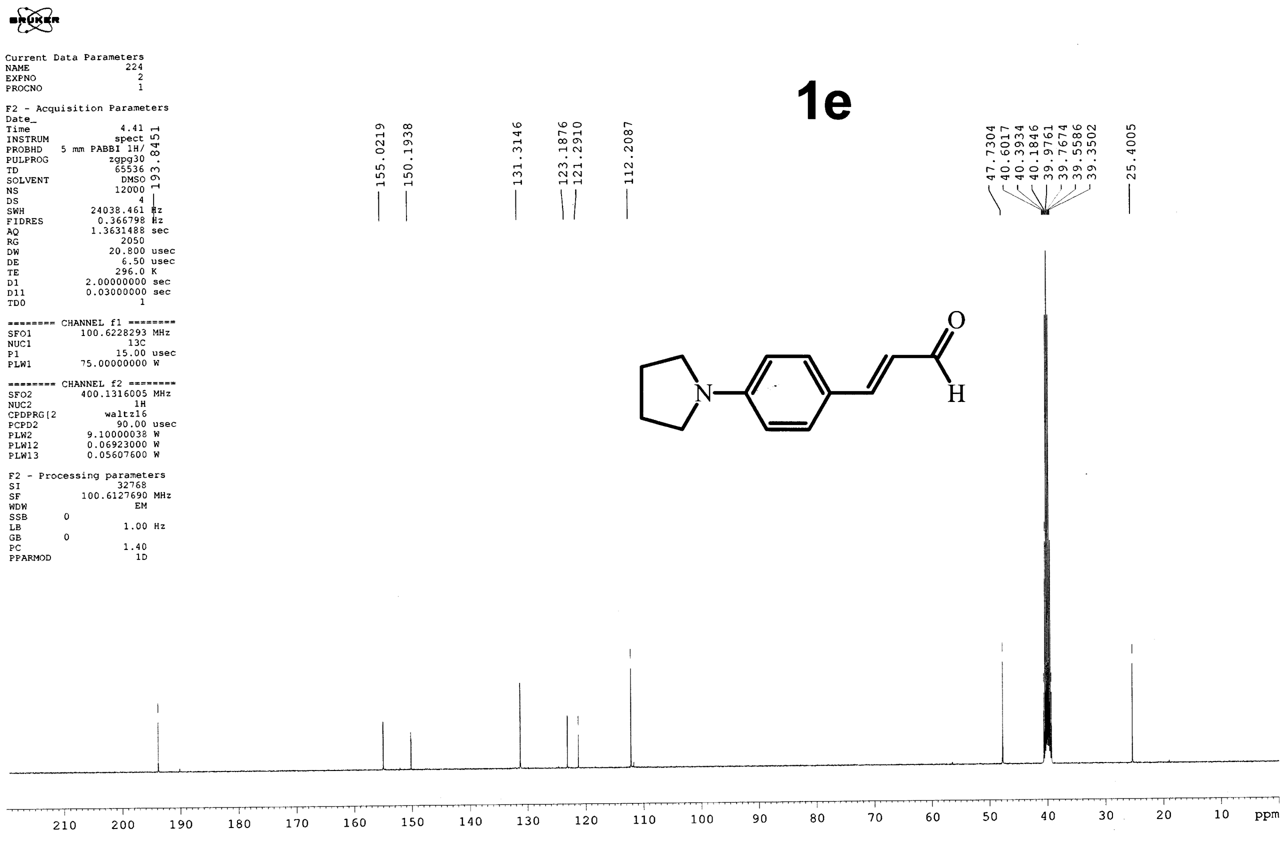

^{1}H NMR spectrum of **1f**

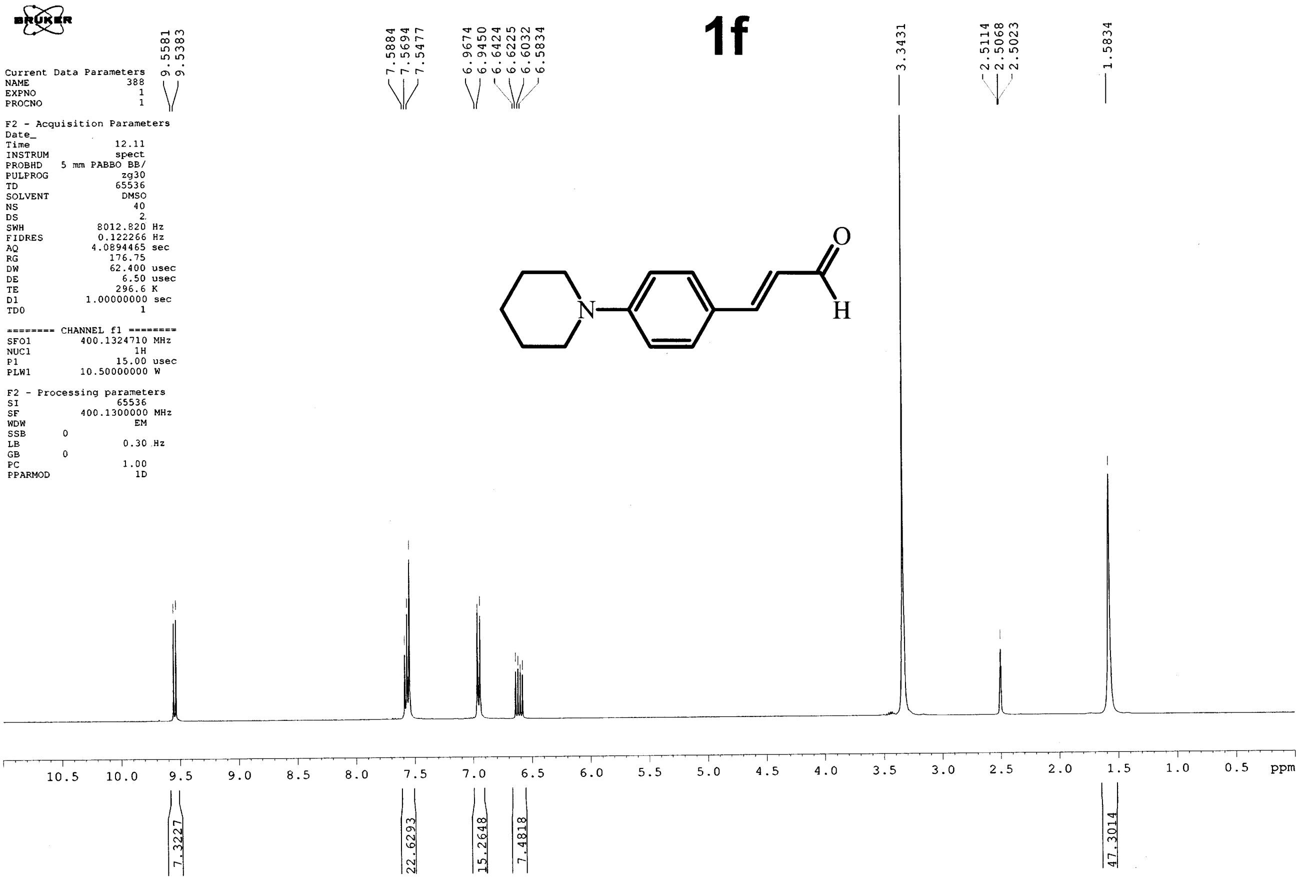

^{13}C NMR spectrum of **1f**

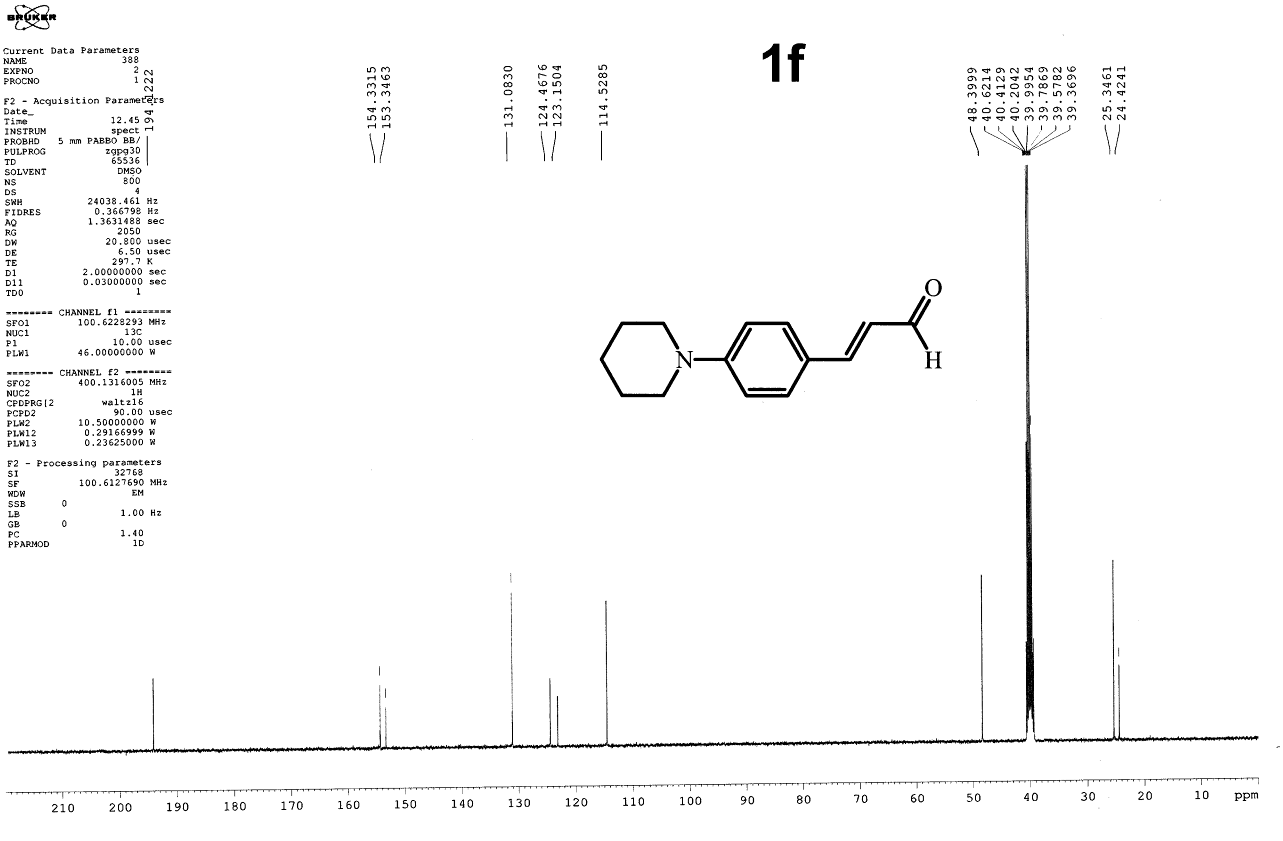

^{1}H NMR spectrum of **1g**

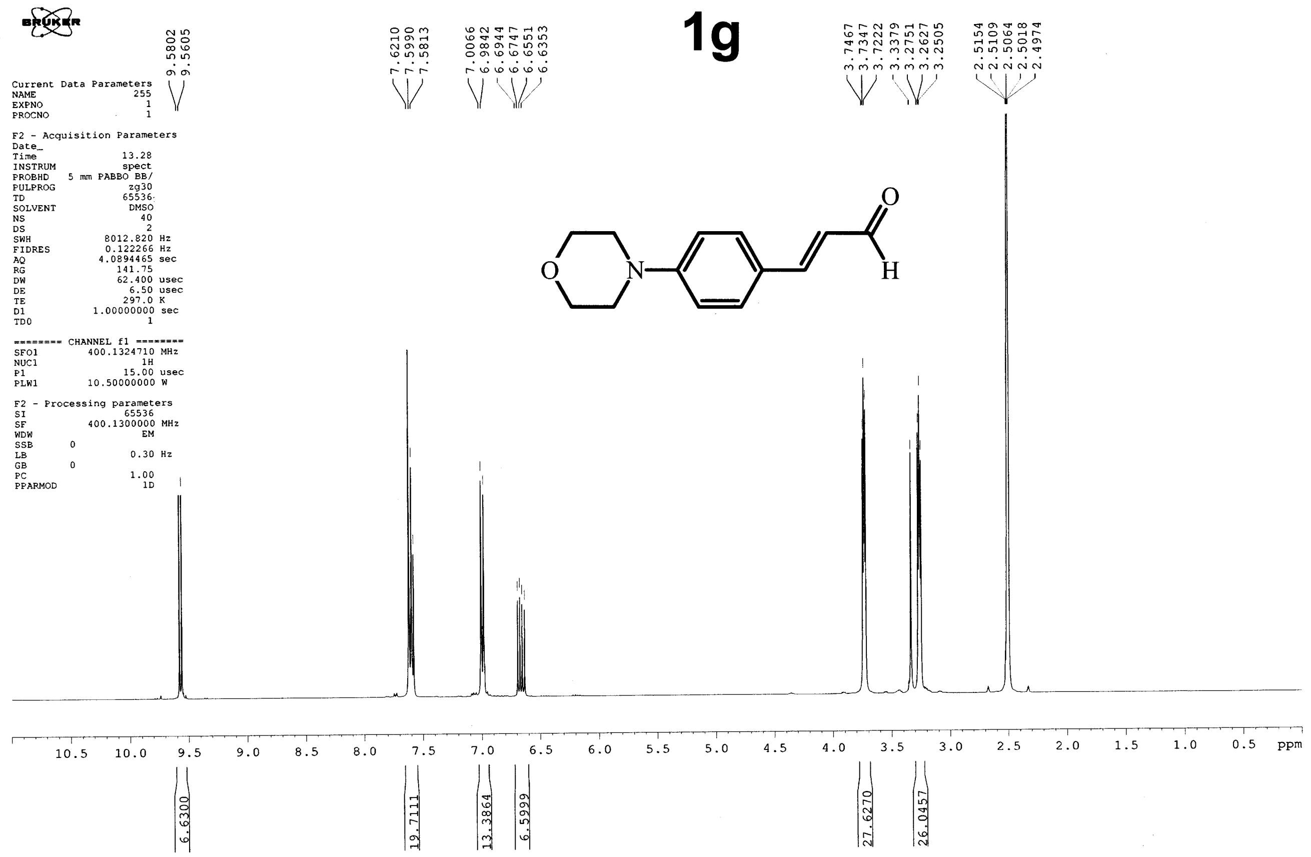

^{13}C NMR spectrum of **1g**

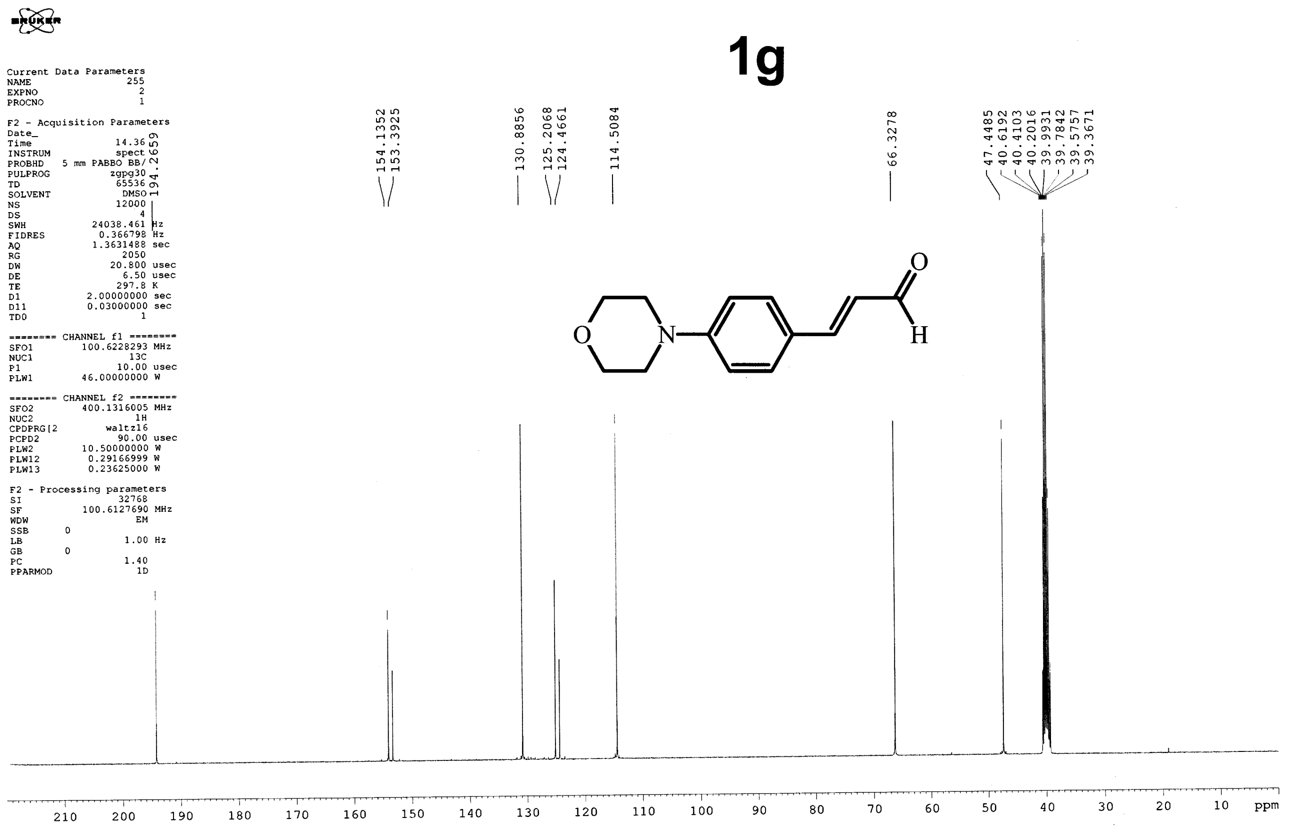

^{1}H NMR spectrum of **1h**

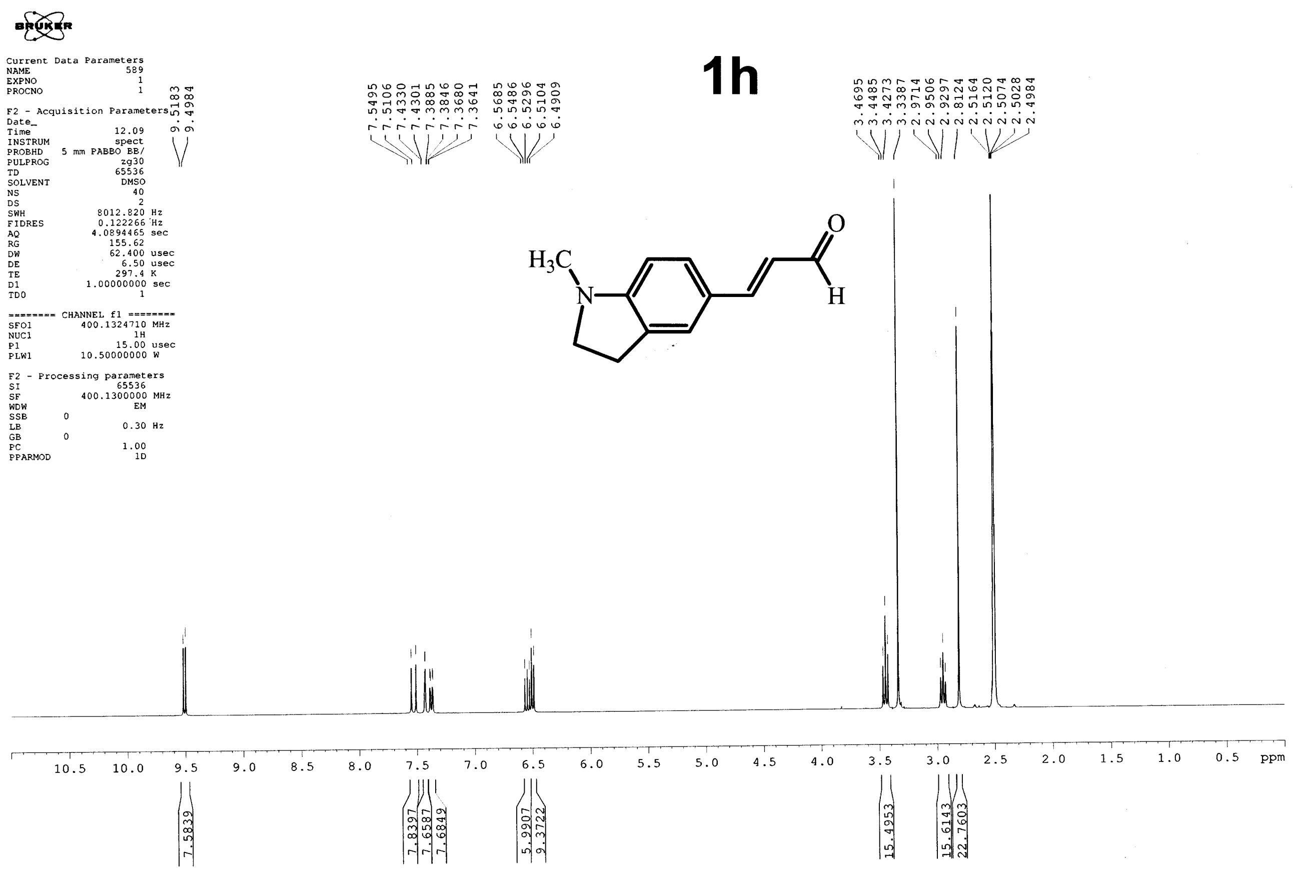

^{13}C NMR spectrum of **1h**

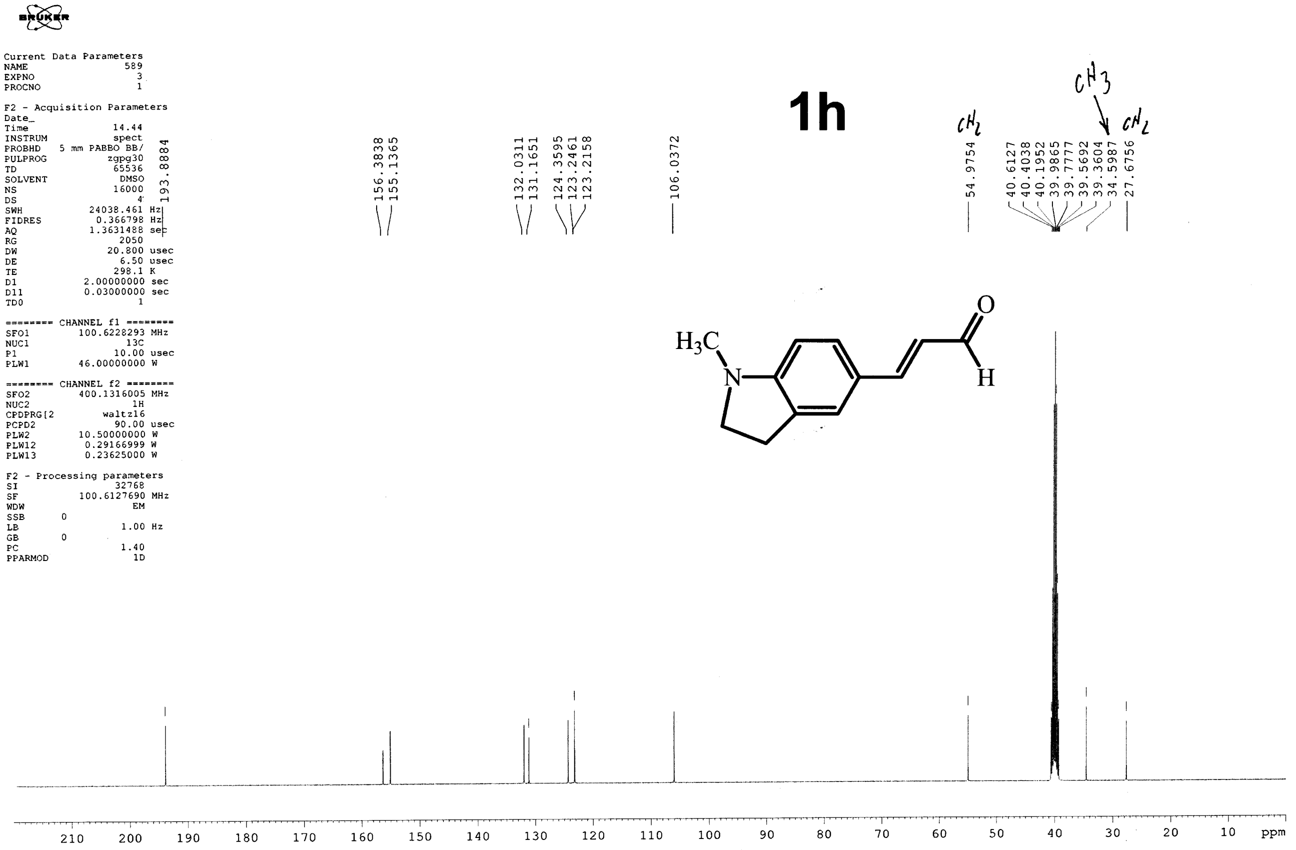

^{1}H NMR spectrum of **1i**

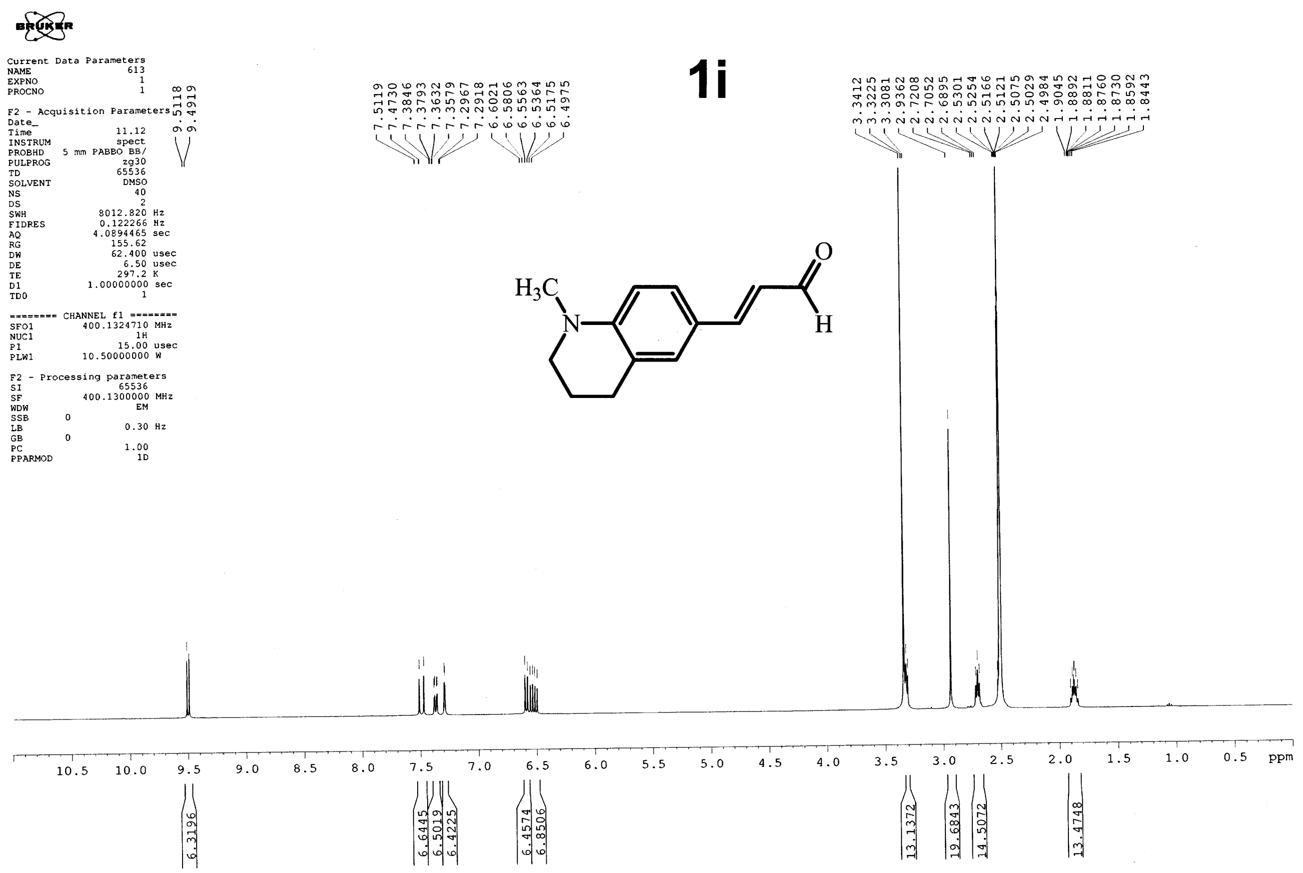

^{13}C NMR spectrum of **1i**

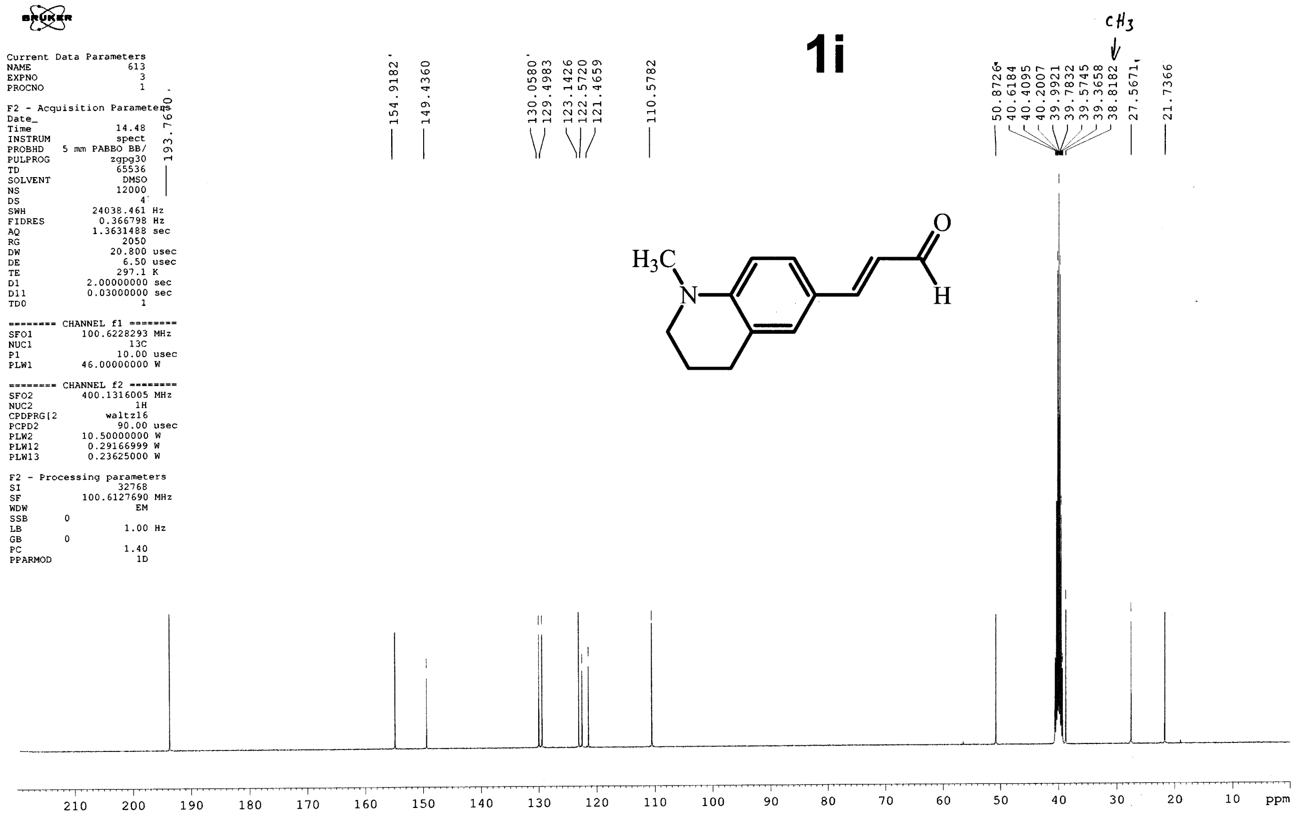

^{1}H NMR spectrum of **1j**

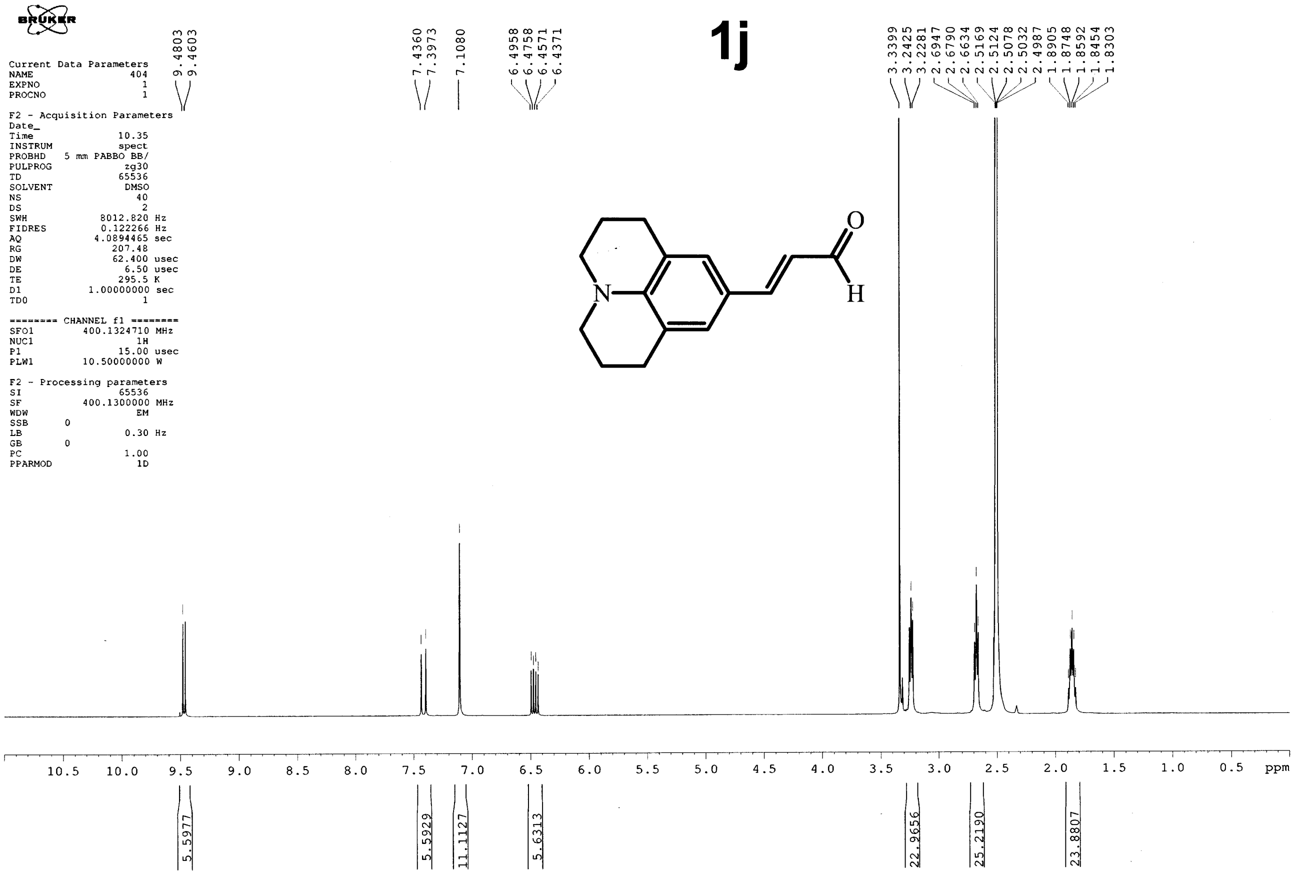

^{13}C NMR spectrum of **1j**

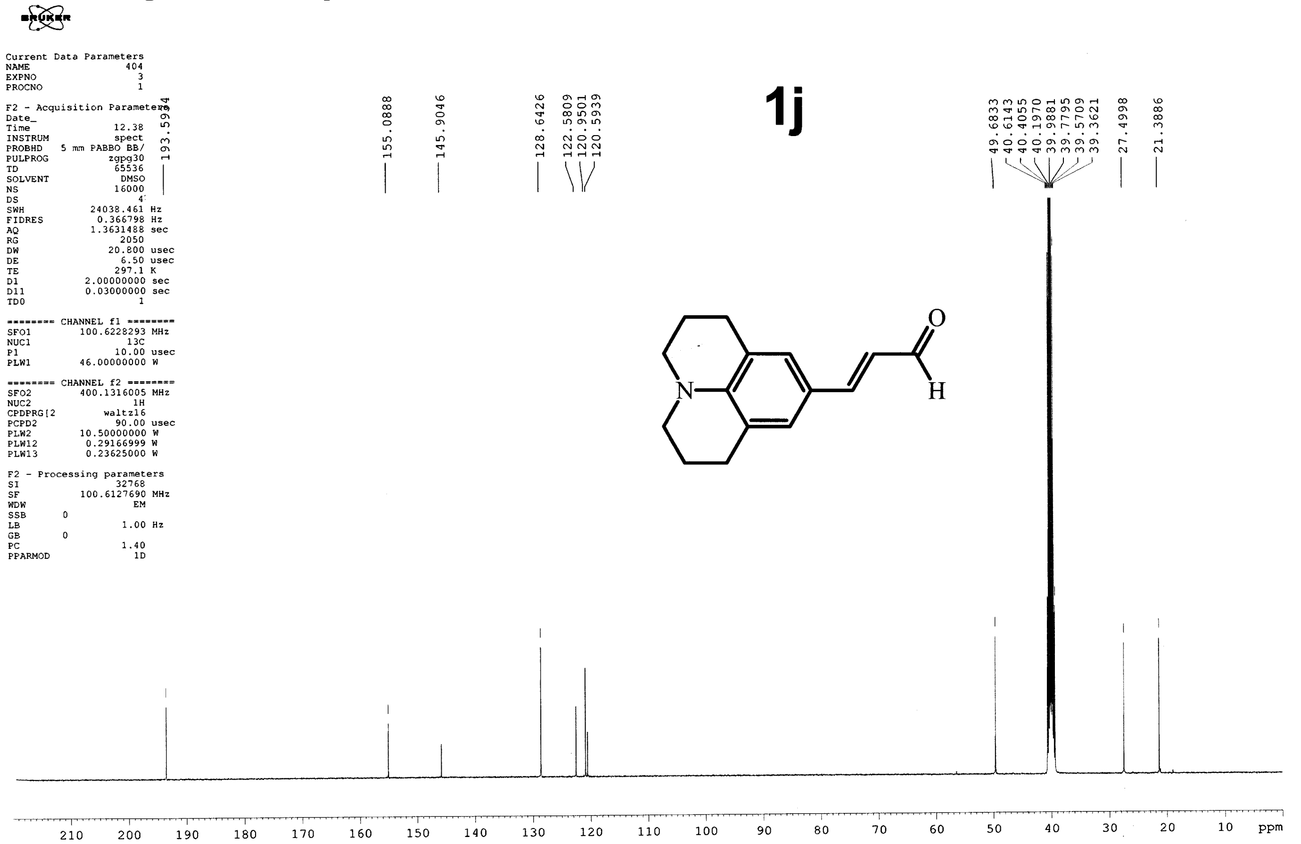

IR spectrum of **1a**

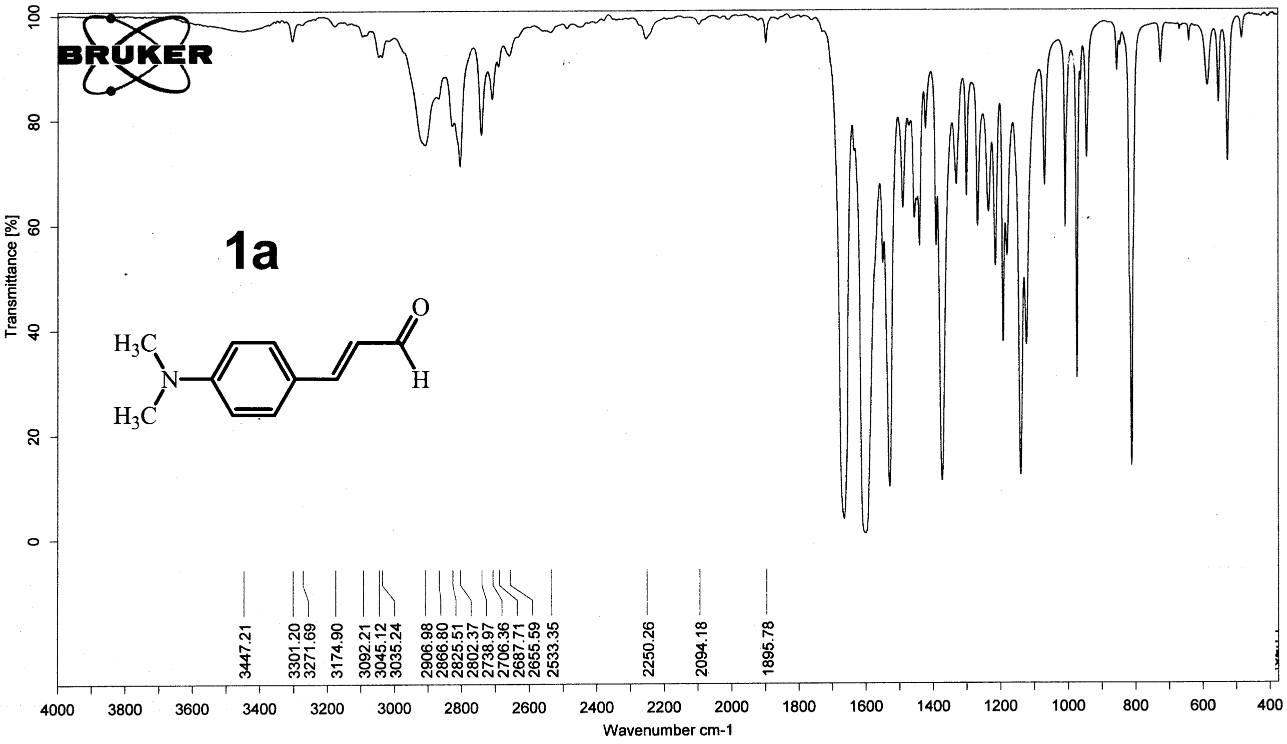

Enlarged spectrum in the range of 1700 - 400 cm^{-1}.

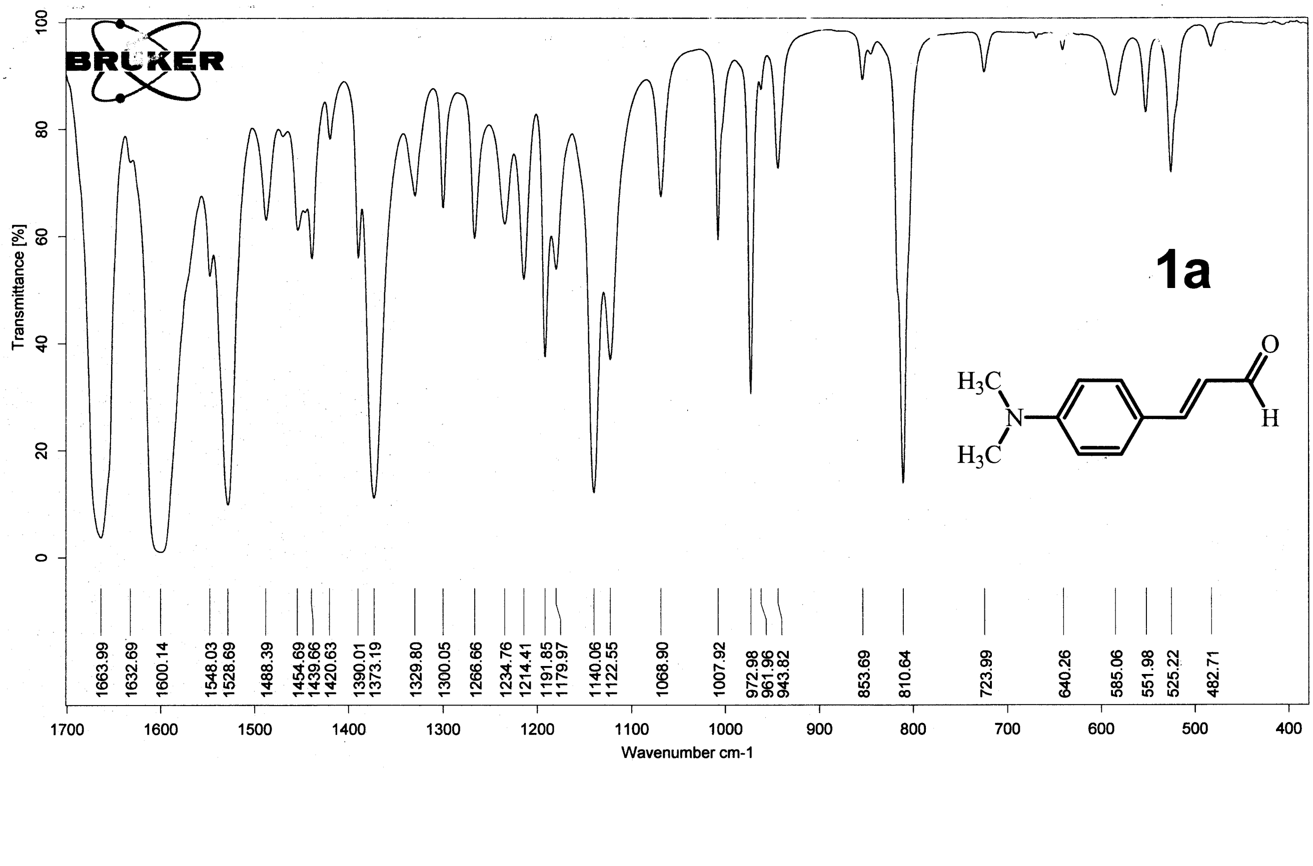

IR spectrum of **1b**

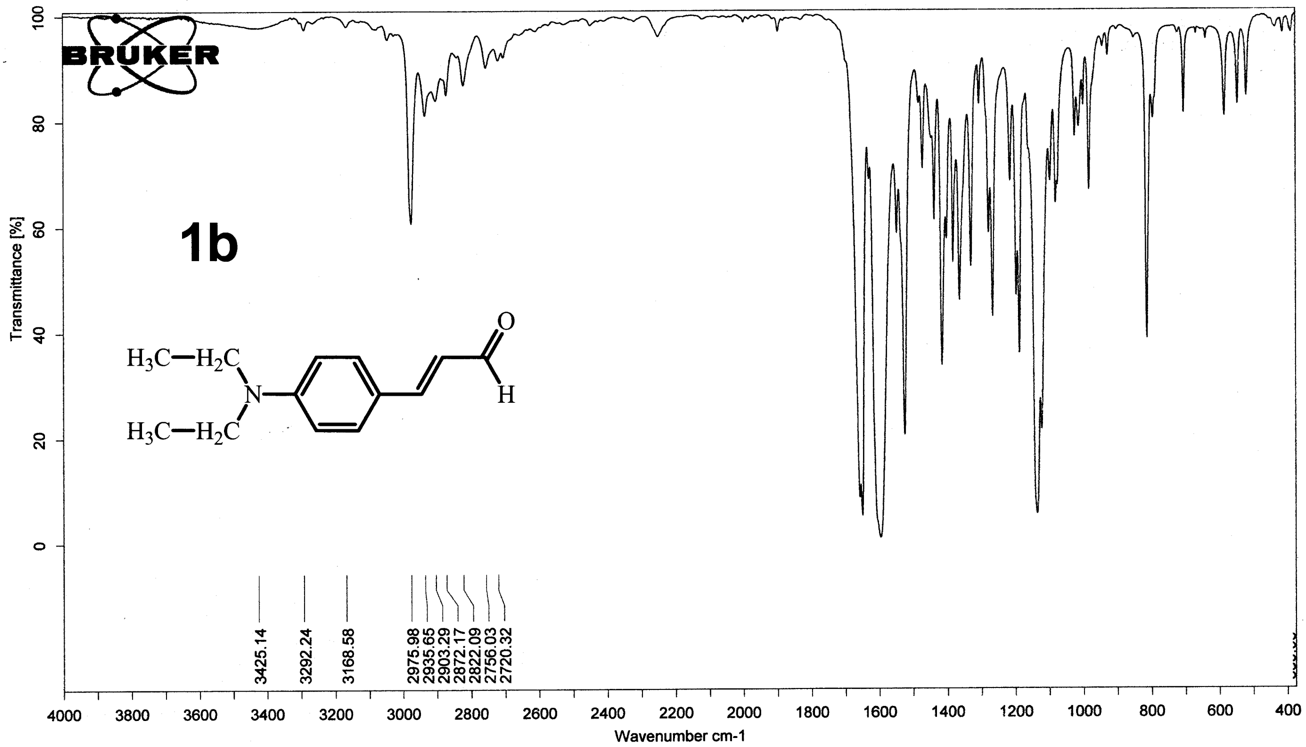

Enlarged spectrum in the range of 1700 - 400 cm^{-1}.

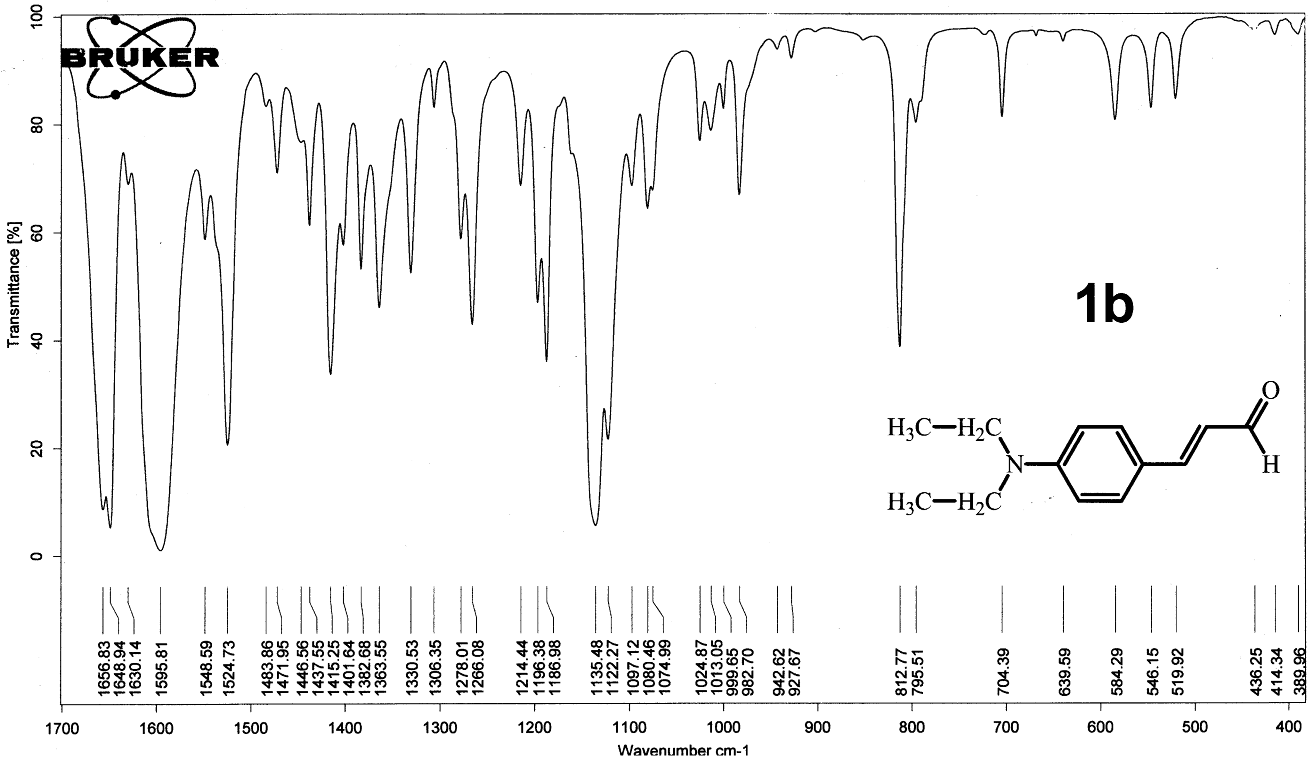

IR spectrum of **1c**

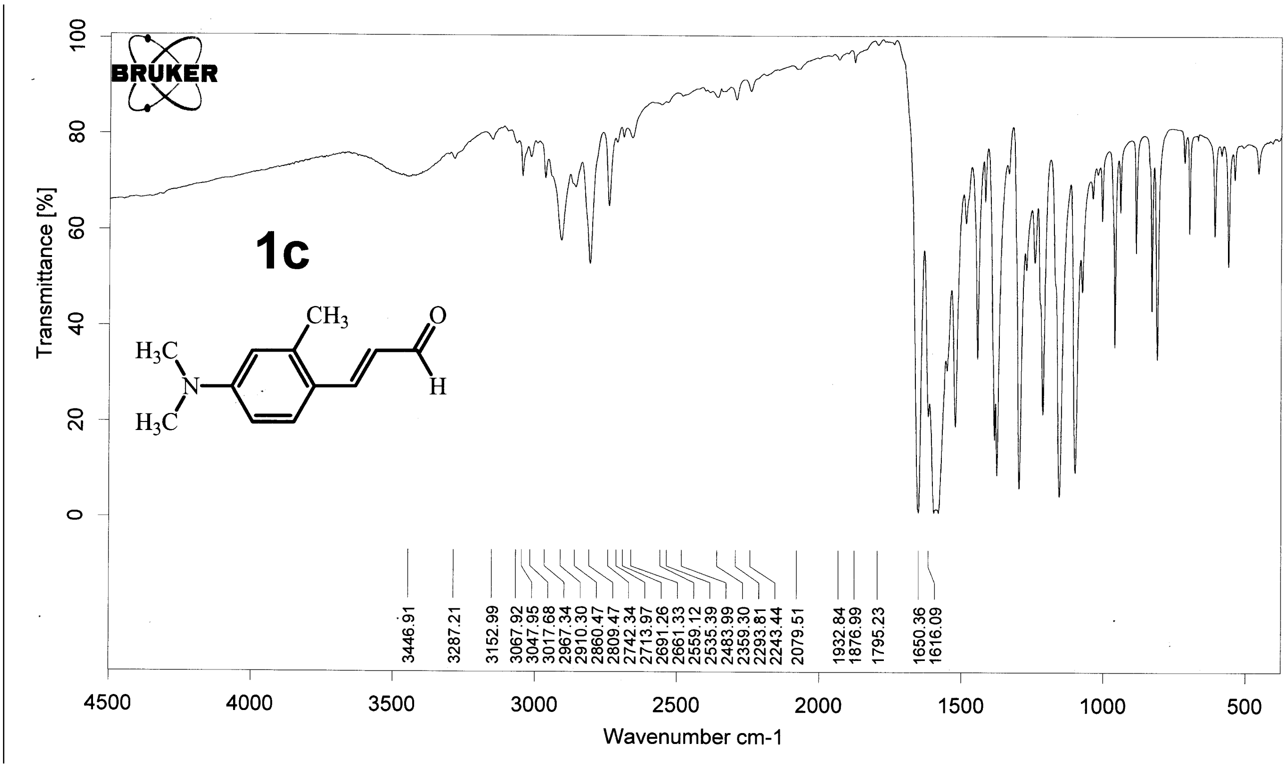

Enlarged spectrum in the range of 1700 - 400 cm^{-1}.

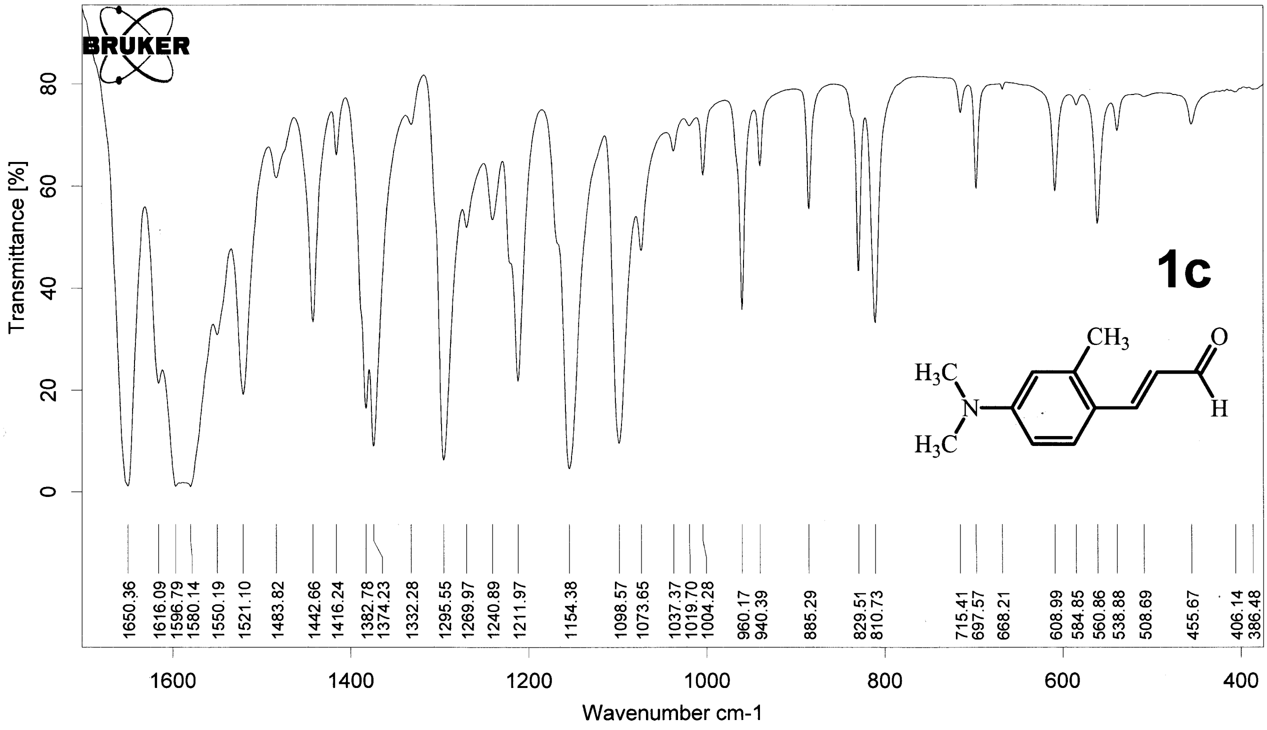

IR spectrum of **1d**

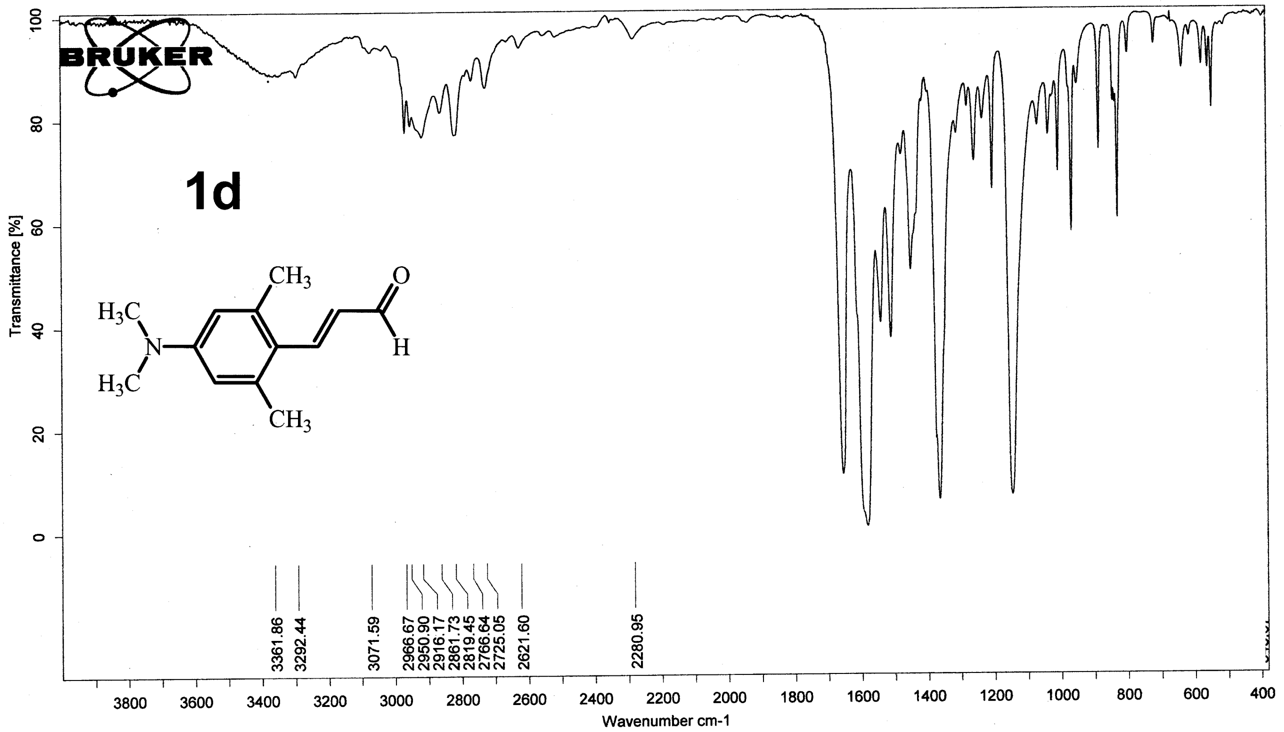

Enlarged spectrum in the range of 1700 - 400 cm^{-1}.

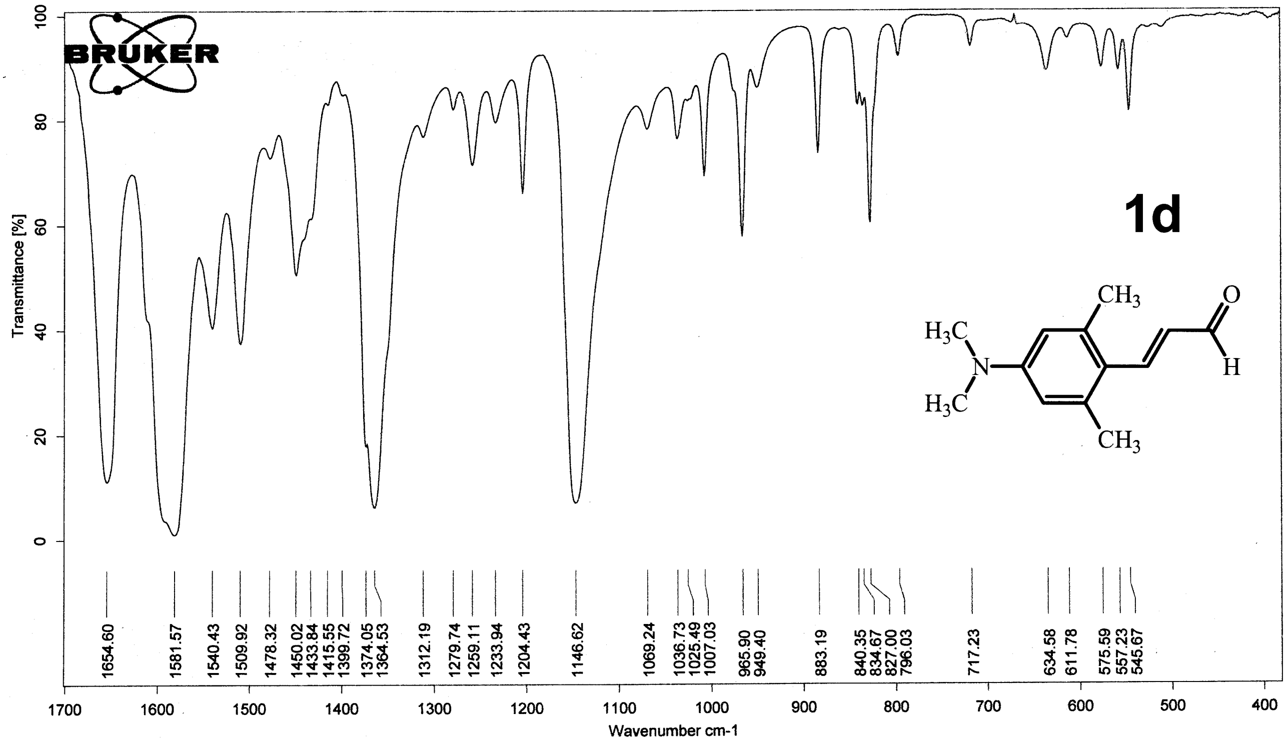

IR spectrum of **1e**

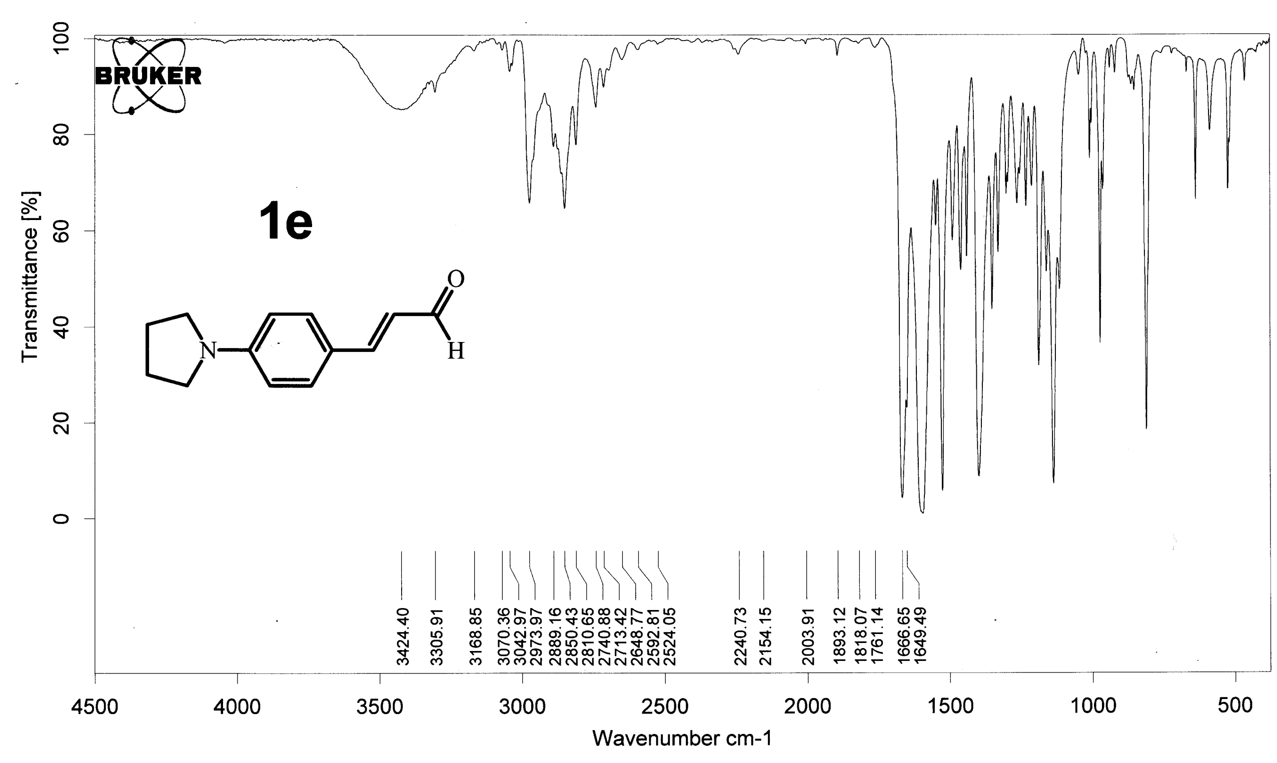

Enlarged spectrum in the range of 1700 - 400 cm^{-1}.

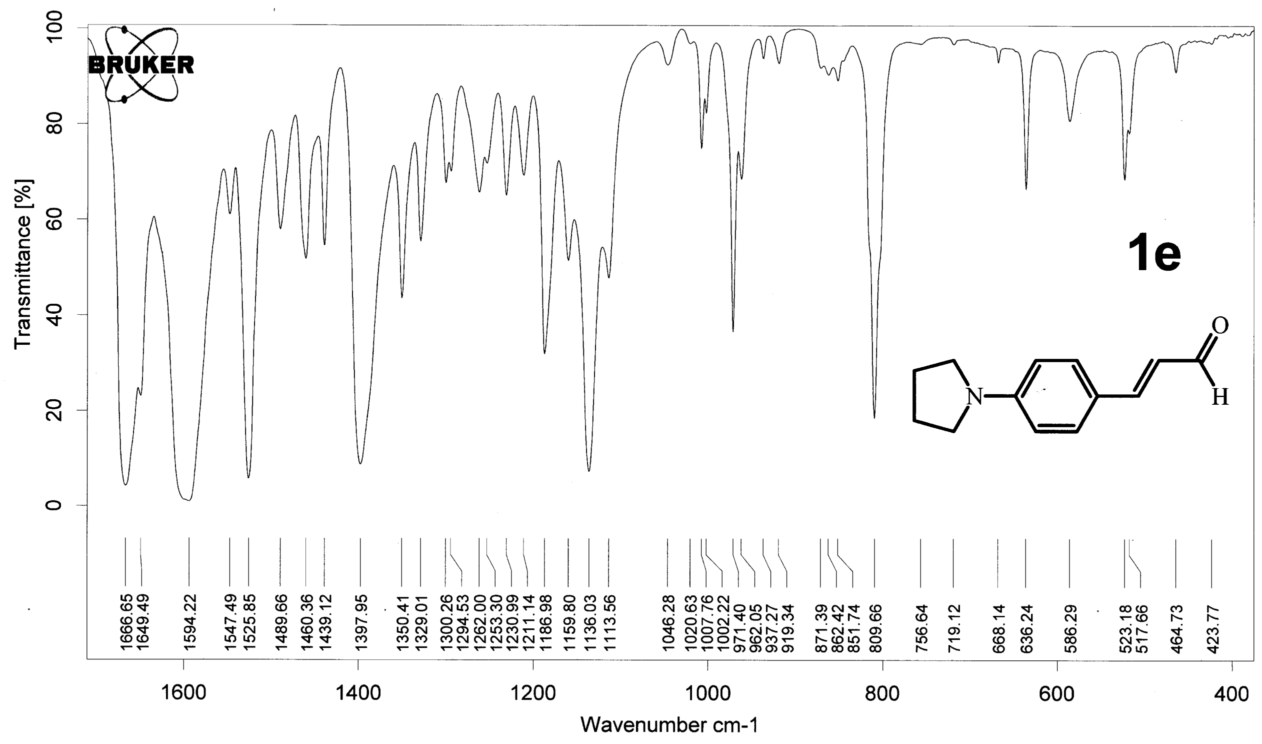

IR spectrum of **1f**

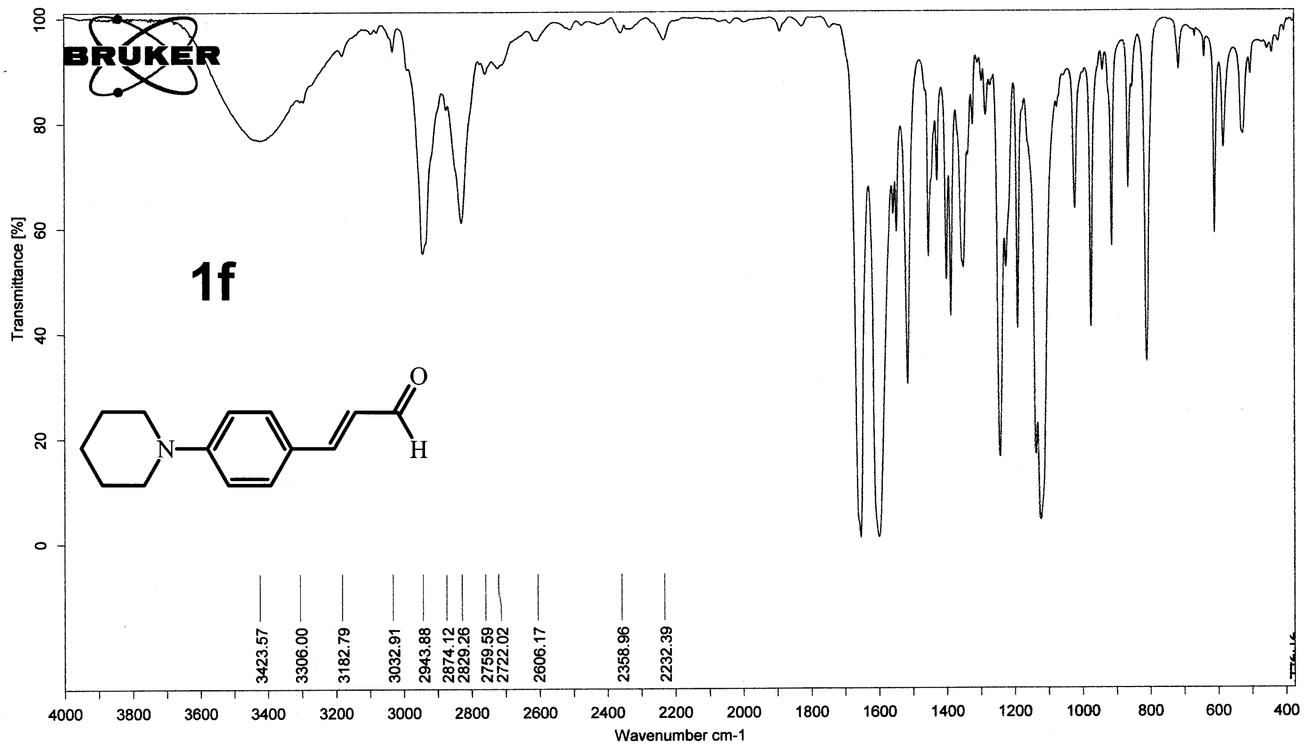

Enlarged spectrum in the range of 1700 - 400 cm^{-1}.

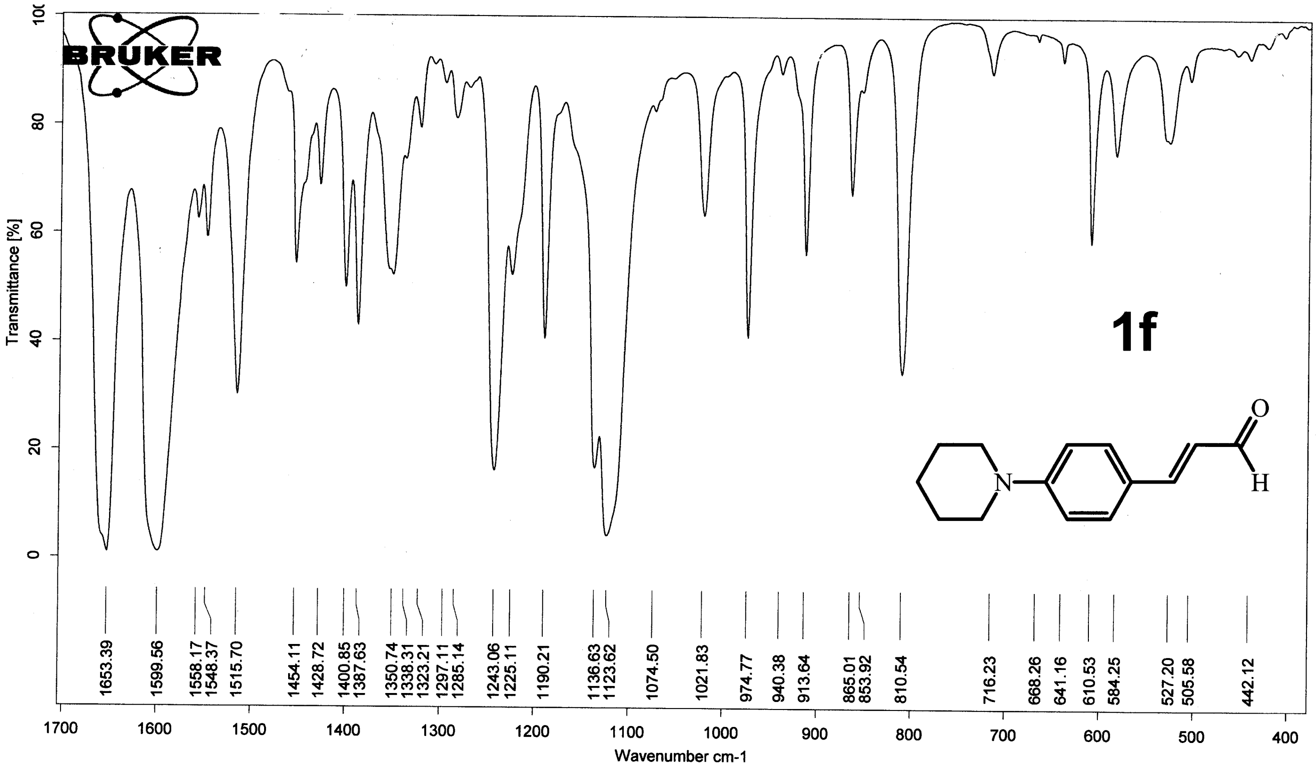

IR spectrum of **1g**

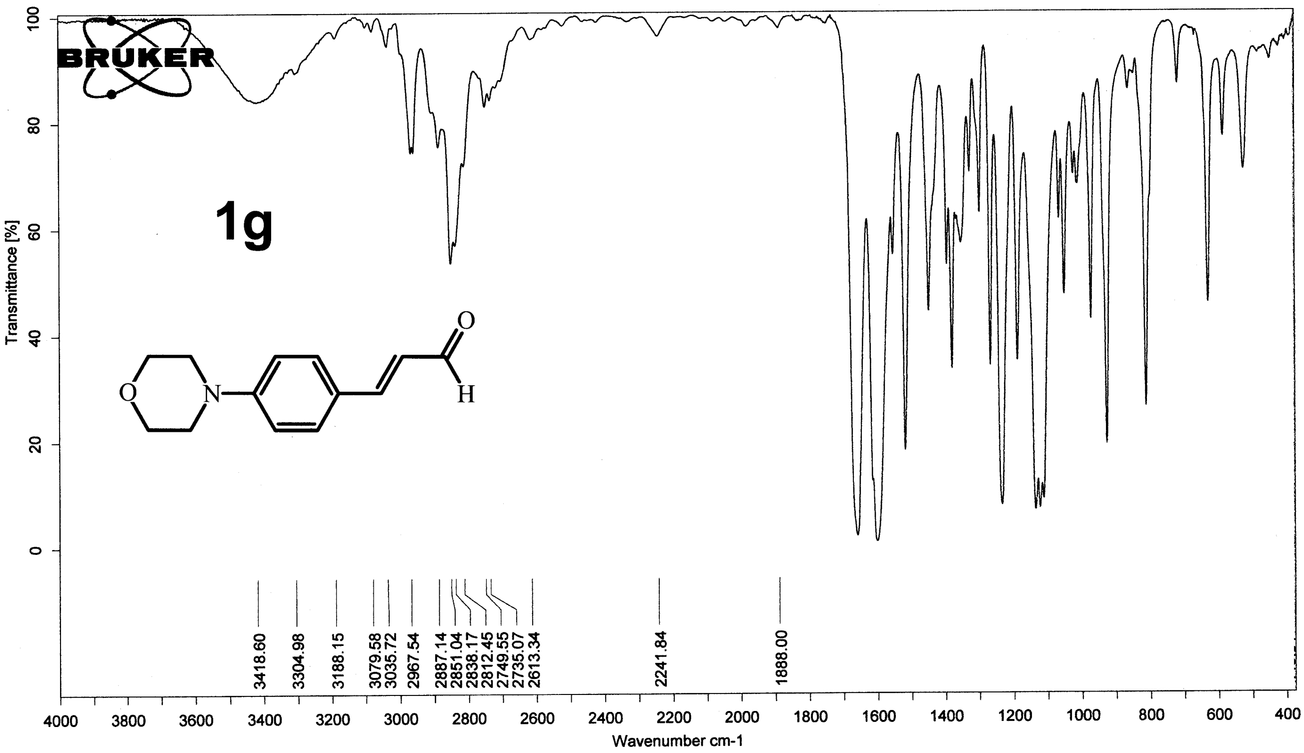

Enlarged spectrum in the range of 1700 - 400 cm^{-1}.

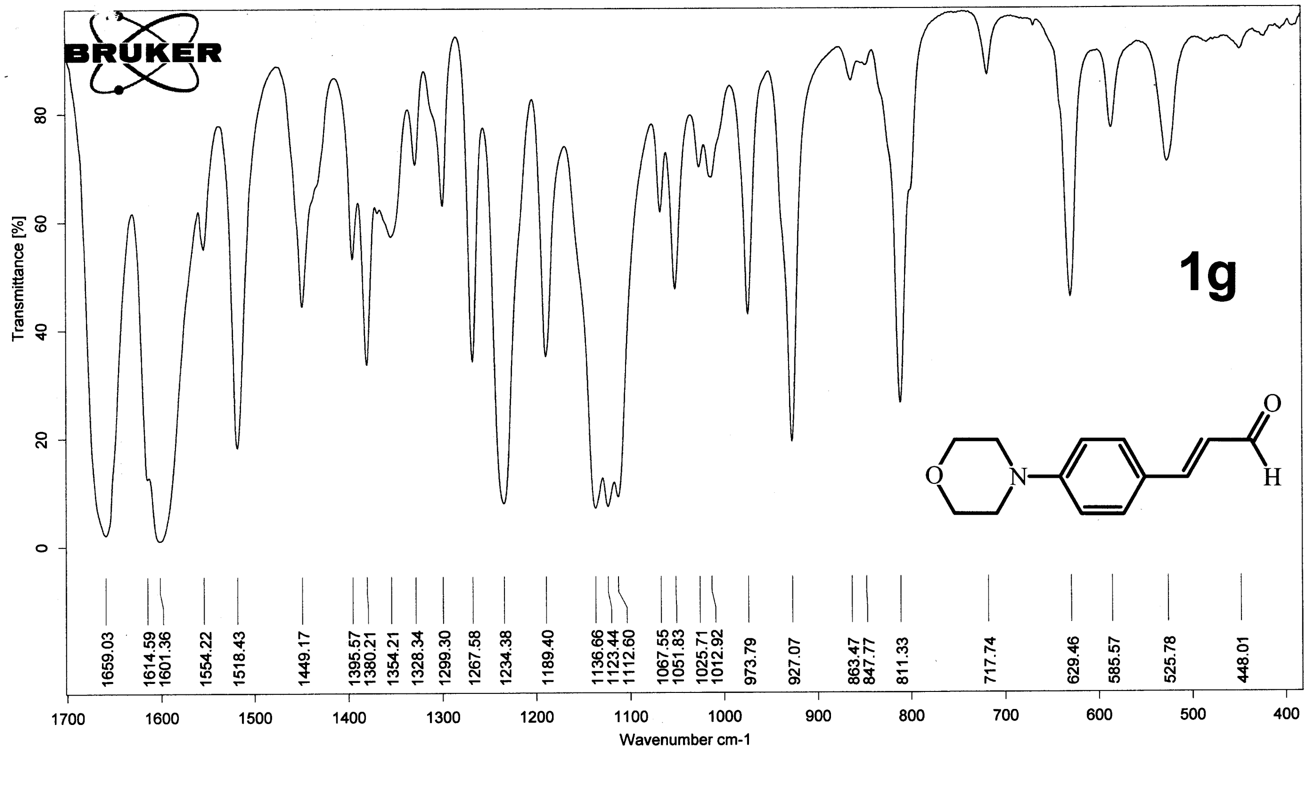

IR spectrum of **1h**

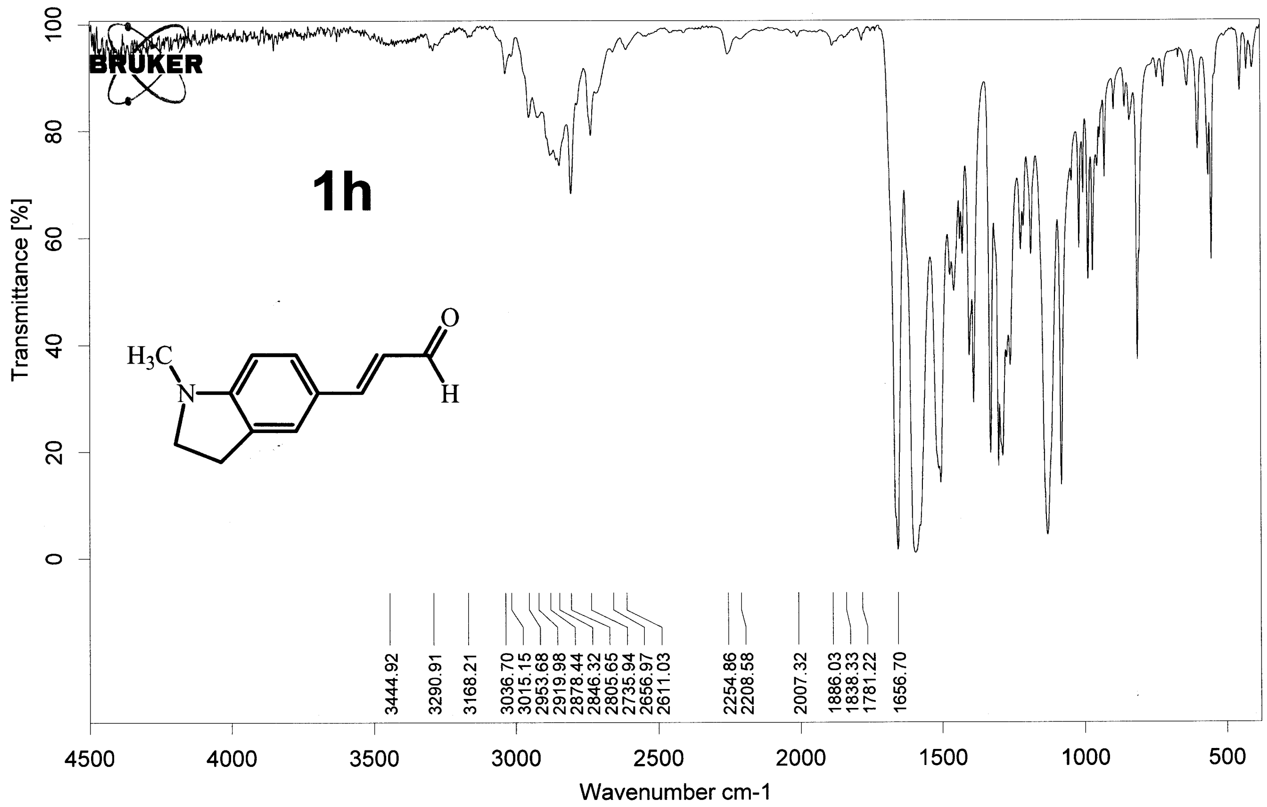

Enlarged spectrum in the range of 1700 - 400 cm^{-1}.

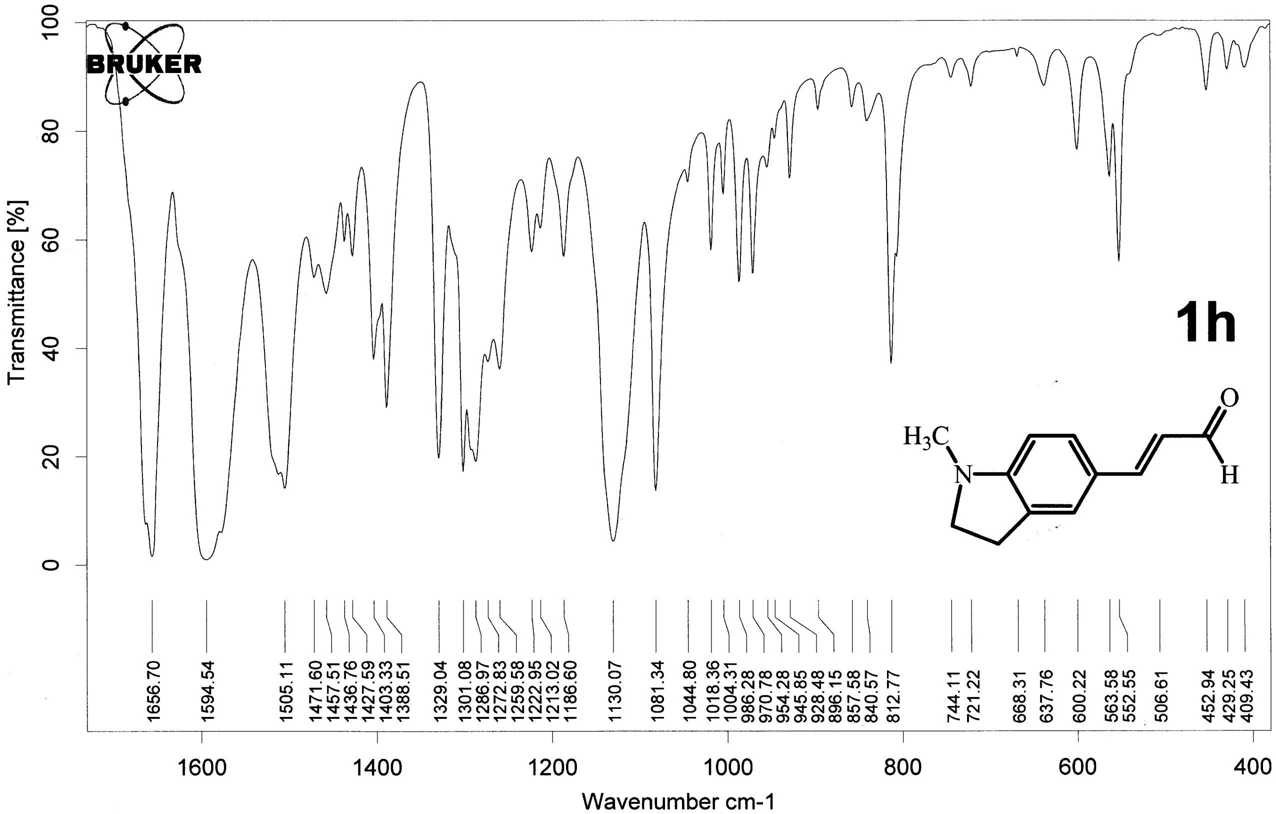

IR spectrum of **1i**

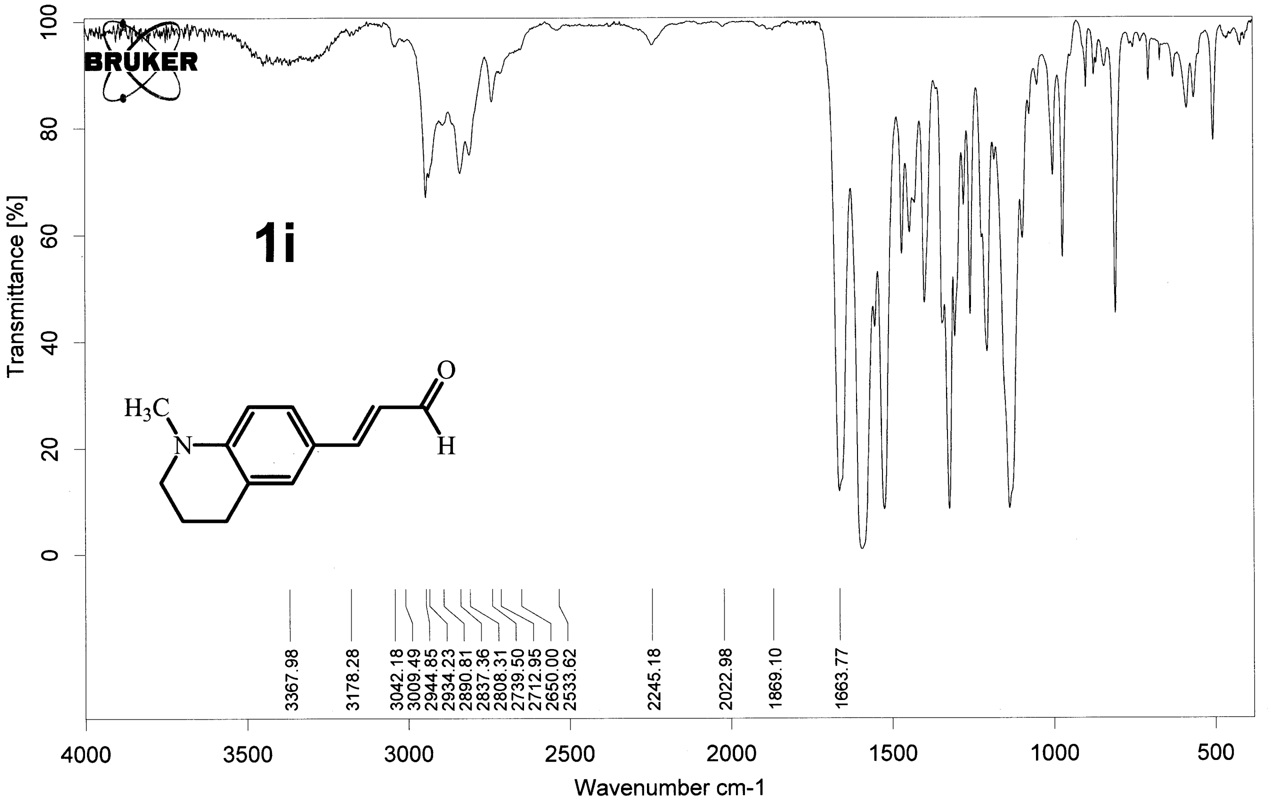

Enlarged spectrum in the range of 1700 - 400 cm^{-1}.

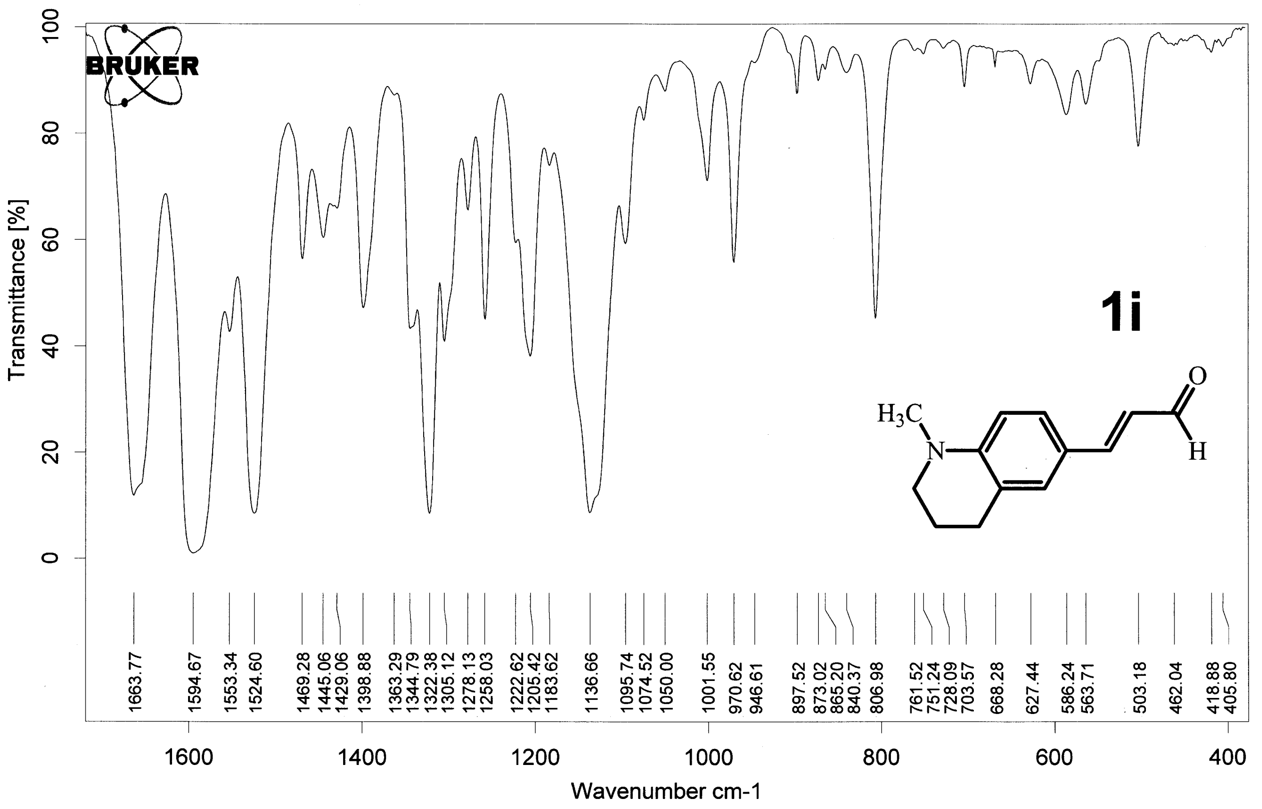

IR spectrum of **1j**

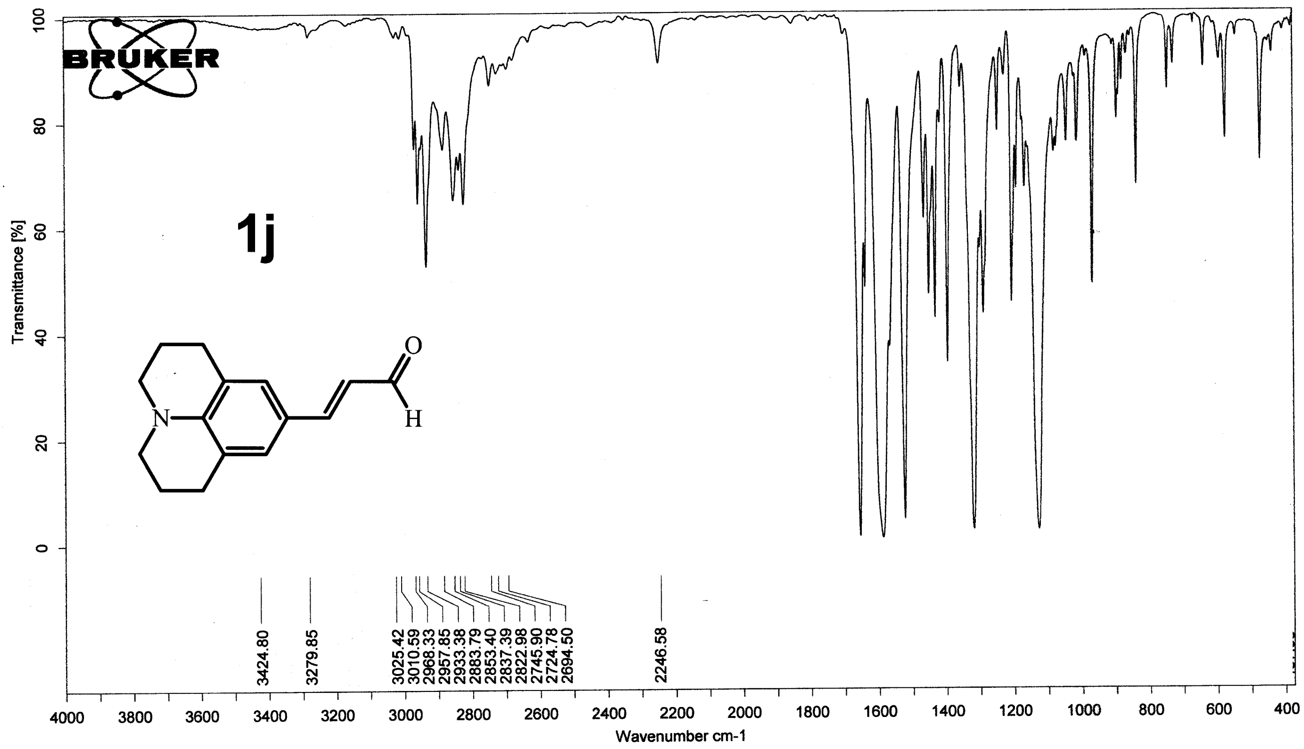

Enlarged spectrum in the range of 1700 - 400 cm^{-1}.

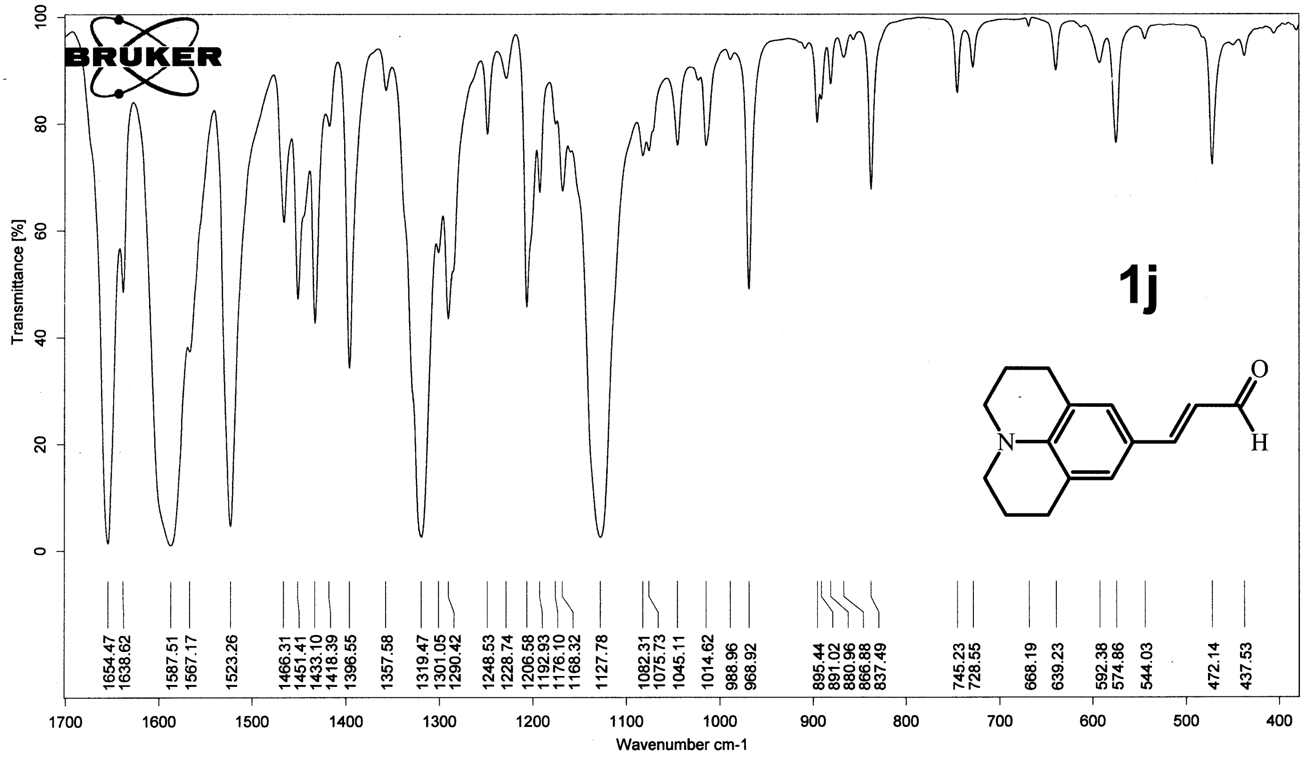

Supplement: Supplementary file 1 [file molecules-26-04360-s001.zip › molecules-1291965-supplementary.pdf]
